# Variants in the vitamin D pathway, serum levels of vitamin D, and estrogen receptor negative breast cancer among African-American women: a case-control study

**DOI:** 10.1186/bcr3162

**Published:** 2012-04-04

**Authors:** Song Yao, Gary Zirpoli, Dana H Bovbjerg, Lina Jandorf, Chi Chen Hong, Hua Zhao, Lara E Sucheston, Li Tang, Michelle Roberts, Gregory Ciupak, Warren Davis, Helena Hwang, Candace S Johnson, Donald L Trump, Susan E McCann, Foluso Ademuyiwa, Karen S Pawlish, Elisa V Bandera, Christine B Ambrosone

**Affiliations:** 1 Department of Cancer Prevention & Control, Roswell Park Cancer Institute, Elm and Carlton Streets, Buffalo, NY 14263, USA; 2 University of Pittsburgh Cancer Institute, University of Pittsburgh, 5150 Centre Avenue, Pittsburgh, PA 15232, USA; 3 Department of Oncological Sciences, Mount Sinai School of Medicine, One Gustave L. Levy Place, New York, NY 10029, USA; 4 Department of Pathology, Roswell Park Cancer Institute, Elm and Carlton Streets, Buffalo, NY 14263, USA; 5 Department of Pharmacology and Therapeutics, Roswell Park Cancer Institute, Elm and Carlton Streets, Buffalo, NY 14263, USA; 6 Department of Medicine, Roswell Park Cancer Institute, Elm and Carlton Streets, Buffalo, NY 14263, USA; 7 New Jersey State Cancer Registry, New Jersey Department of Health & Senior Services, 369 South Warren Street, Trenton, NJ 08608, USA; 8 The Cancer Institute of New Jersey, Robert Wood Johnson Medical School, 125 Paterson Street, New Brunswick, NJ 08901, USA

## Abstract

**Introduction:**

American women of African ancestry (AA) are more likely than European Americans (EA) to have estrogen receptor (ER)-negative breast cancer. 25-hydroxyvitamin D (25OHD) is low in AAs, and was associated with ER-negative tumors in EAs. We hypothesized that racial differences in 25OHD levels, as well as in inherited genetic variations, may contribute, in part, to the differences in tumor characteristics.

**Methods:**

In a case (*n *= 928)-control (*n *= 843) study of breast cancer in AA and EA women, we measured serum 25OHD levels in controls and tested associations between risk and tag single nucleotide polymorphisms (SNPs) in *VDR*, *CYP24A1 *and *CYP27B1*, particularly by ER status.

**Results:**

More AAs had severe vitamin D deficiency (< 10 ng/ml) than EAs (34.3% vs 5.9%), with lowest levels among those with the highest African ancestry. Associations for SNPs differed by race. Among AAs, *VDR *SNP rs2239186, associated with higher serum levels of 25OHD, decreased risk after correction for multiple testing (OR = 0.53, 95% CI = 0.31-0.79, *p *by permutation = 0.03), but had no effect in EAs. The majority of associations were for ER-negative breast cancer, with seven differential associations between AA and EA women for *CYP24A1 *(*p *for interaction < 0.10). SNP rs27622941 was associated with a > twofold increased risk of ER-negative breast cancer among AAs (OR = 2.62, 95% CI = 1.38-4.98), but had no effect in EAs. rs2209314 decreased risk among EAs (OR = 0.38, 95% CI = 0.20-0.73), with no associations in AAs. The increased risk of ER-negative breast cancer in AAs compared to EAs was reduced and became non-significant (OR = 1.20, 95% CI = 0.80-1.79) after adjusting for these two *CYP24A1 *SNPs.

**Conclusions:**

These data suggest that genetic variants in the vitamin D pathway may be related to the higher prevalence of ER-negative breast cancer in AA women.

## Introduction

American women of African ancestry (AA) are more likely to develop breast cancer at a younger age than those with European ancestry (EA) and are more likely to have tumors with aggressive characteristics, including high histological grade, negative estrogen receptor (ER) status, and basal-like - ER^- ^and/or progesterone receptor (PR)^-^, HER2^-^, and cytokeratin 5/6^+ ^and/or HER1^+ ^-features [1,2]. The reasons for these racial disparities are unknown.

It is clear that, among geographically diverse populations, certain genotypic and phenotypic characteristics may be selected for in response to local environmental pressures [3]. Skin pigmentation, the primary factor that provides protection from ultraviolet (UV) radiation, is correlated with latitude, and dark skin pigmentation is likely to be the original ancestral trait in humans. Migrations to Europe and Asia eventually gave rise to decreased pigmentation and lighter skin [4-6]. As much as 90% of vitamin D is derived from sun exposure, but high skin melanin concentration prevents penetration of UVB light and compromises synthesis efficiency by 10 to 50 times [7]. Although high pigmentation would reduce absorption of vitamin D, intense sun exposure in sub-Saharan Africa would compensate. However, in high-latitude areas where UVB intensity is low and where more time may be spent indoors (particularly in winter), vitamin D deficiency may result among individuals with higher skin pigmentation. Indeed, in the US, the prevalence of 25-hydroxyvitamin D (25OHD) of less than 15 ng/mL is almost 10 times higher in AA than in EA women [8], and the prevalence of severe vitamin D deficiency (< 10 ng/mL) among AAs was 29% in 2001 to 2004 [9]. In contrast, in Guinea-Bissau, the average 25OHD levels in healthy Africans were 34 ng/mL, and the prevalence of severe vitamin D deficiency was as low as 1% [10].

Endogenous 25OHD may also be affected by variability in metabolic pathways, and synthesis and metabolism catalyzed by two major enzymes, 1α-hydroxylase and 24-hydroxylase, which are encoded by *CYP27B1 *and *CYP24A1*, respectively. Binding of vitamin D to the vitamin D receptor (VDR) activates or suppresses gene transcription, depending on the type of response elements [11], and genetic variability in the above genes, known to differ by ancestry [12], is likely to affect vitamin D signaling.

Laboratory, preclinical, and clinical findings support the hypothesis that low levels of vitamin D are related to breast cancer risk. In the human mammary gland, VDR is expressed in all cell types [13], and vitamin D treatment inhibits breast cancer cell proliferation, induces cell apoptosis, and prevents carcinogenesis in rodent models [14,15]. However, epidemiologic evidence for associations between vitamin D and breast cancer risk is considered 'limited' [16], and one randomized trial showed little impact of vitamin D supplementation on breast cancer incidence [17]. These inconclusive findings could be due to tumor heterogeneity, which implies that the effects of vitamin D may only present in specific breast cancer subtypes. In fact, *Vdr *knockout mice were more likely than their wild-type littermates to develop *Er/Pr*^- ^tumors [18]. Consistent with results from these preclinical studies, some epidemiologic studies have also indicated that effects of vitamin D may be strongest for breast cancers with poor prognostic characteristics and that lower serum 25OHD levels are found among women with ER^- ^compared with ER^+ ^tumors [19-21]. Recently, we reported lower levels of serum 25OHD in women with high- versus low-grade breast tumors and in women with triple-negative versus luminal A breast tumors [22].

Here, we examined levels of 25OHD in AA and EA women without breast cancer in relation to self-reported race as well as ancestry, which was estimated by using ancestry informative markers (AIMs). 25OHD levels in women with breast cancer could be a result of disease processes, and some samples were obtained after chemotherapy was initiated; thus, we did not compare serum levels of 25OHD between cases and controls. Instead, we evaluated variants in vitamin D activity and major metabolism (*VDR*, *CYP27B1*, and *CYP24A1*) in relation to breast cancer risk, particularly in relation to self-reported race and estrogen receptor status. We also tested whether vitamin D-related genetic variants could explain, in part, the higher prevalence of ER^- ^breast cancer among AA women.

## Materials and methods

### Study population

The Women's Circle of Health Study (WCHS) is an ongoing study designed specifically to examine the role of genetic and non-genetic factors in early/aggressive breast cancer in AA and EA women. Study design, enrollment, and collection of data and biospecimens have been described in detail previously [23]. Briefly, women with diagnosed incident breast cancer were identified through both hospital-based case ascertainment in targeted hospitals that had large referral patterns of AAs in four boroughs of the metropolitan New York City area and population-based case ascertainment in seven counties in New Jersey through the New Jersey State Cancer Registry. The eligibility criteria for cases were the following: self-identified AA and EA women, 20 to 75 years of age at diagnosis, no previous history of cancer other than non-melanoma skin cancer, recent diagnosis of primary, histologically confirmed breast cancer, and English-speaking. EA women with breast cancer, more prevalent in the catchment area than AA cases, were randomly selected for recruitment and were matched by age and county to AA cases. Controls who did not have a history of diagnosis of any cancer other than non-melanoma skin cancer and who were living in the same area as cases were identified through random digit dialing and were matched to cases by self-reported race and 5-year age categories. After agreement to participate was obtained, in-person interviews were conducted to complete informed consent and to query participants on a number of potential risk factors, including medical history, family history of cancer, diet, physical activity, and other lifestyle factors. Anthropometric measures were taken, and biospecimens were collected. Blood samples were initially collected, but owing to logistical and cost constraints, we transitioned to saliva samples after the enrollment of approximately 850 participants. Permission to obtain pathology data, including ER status, and tumor tissue blocks was included in the informed consent form. The participation rates were 80.2% and 53.4% for AA cases and controls, respectively, and 80.0% and 48.9% for EA cases and controls, respectively. This study was approved by the institutional review boards of the Roswell Park Cancer Institute, the Cancer Institute of New Jersey, the Mount Sinai School of Medicine, and the participating hospitals in New York City. At the time of the genotyping (April 2010), DNA and data were available for 553 AA cases and 466 AA controls and 383 EA cases and 382 EA controls from the WCHS.

### Selection of multi-population tag single-nucleotide polymorphisms

We used a two-step approach to select a set of multi-population tag single-nucleotide polymorphisms (SNPs) that represent common genetic variations - minor allele frequency of at least 0.05 - in *VDR *in both AA and EA populations. First, 122 SNPs in the *VDR *region plus 15-kb regions from both 3' and 5' ends were selected from HapMap [24] and other resequencing projects by using the Genome Variation Server at SeattleSNPs [25]. These SNPs were then genotyped in 60 AA and 60 EA controls. With the TAGster program [26], 49 multi-population tag SNPs were subsequently selected for genotyping in the WCHS. Also among those *VDR *variants finally selected were commonly studied SNPs, including Cdx2 (rs11568820), Fok1 (rs2228570), Bsm1 (rs1544410), Apa1 (rs7975232), and Taq1 (rs731236). For *CYP24A1*, 15 multi-population tag SNPs were selected by using publicly available CEU (Utah residents with ancestry from Northern and Western Europe) and YRI (Yoruba in Ibadan) genotype data from HapMap. For *CYP27B1*, only one SNP had a minor allele frequency of at least 0.05 in the dbSNP database and was thus included. To control for potential bias due to population admixture and to examine serum 25OHD levels in relation to ancestry, a panel of 108 AIMs that have been shown to be effective in correcting for admixture in case-control studies [27] was chosen.

### Genotyping

DNA was extracted from blood samples by using FlexiGene™ DNA kits (Qiagen, Valencia, CA, USA) in accordance with the instructions of the manufacturer and from saliva collected in Oragene™ kits (DNA Genotek Inc., Kanata, ON, Canada). Genomic DNA was evaluated and quantified by a Nanodrop UV spectrometer (Thermo Fisher Scientific Inc., Wilmington, DE, USA) and a PicoGreen-based fluorometric assay (Molecular Probes, now part of Invitrogen Corporation, Carlsbad, CA, USA) and stored at -80°C until analysis. Selected tag SNPs and AIMs were genotyped by an Illumina GoldenGate assay (Illumina Inc., San Diego, CA, USA) at the Genomics Core Facility at the Roswell Park Cancer Institute. Five percent duplicates and two sets of in-house trio samples were included for quality control purposes. The average successful genotyping rate for each sample and each SNP was at least 99%, and no SNPs violated Hardy-Weinberg equilibrium in controls or mendelian inheritance. Clustering plots of SNPs that were significant in the statistical analysis were manually re-inspected *post hoc *to ensure that the calls were robust.

### Measurement of serum levels of 25OHD in WCHS controls

Serum samples were available from 242 AA and 187 EA women in the control group and were used to measure levels of 25OHD by immunochemiluminometric assay. The assay coefficient of variation was 10.3%.

### Statistical analysis

#### Estimation of ancestry and comparison of serum levels of 25OHD

Individual ancestral proportions for EA and AA were estimated with the Bayesian Markov chain Monte Carlo clustering algorithm implemented in STRUCTURE 2.3 [28]. We included publicly available genotypes from the YRI and CEU ancestral populations, and the program was run multiple times assuming K = 2 underlying ancestries. Women with more than 85% of genomic race other than the self-identified race were excluded from the analysis (*n *= 13); therefore, 547 AA cases and 461 AA controls and 381 EA cases and 382 EA controls were included in the final analyses for SNPs and breast cancer risk. There were no exclusions due to conflicting self-report and marker ancestry in the serum analyses performed in controls only. To compare serum levels of 25OHD between AA and EA women, least squares means and standard errors were calculated with adjustment for age, body mass index (BMI), and season of blood collection (four seasons). To test genotype-phenotype correlations between SNPs and 25OHD levels in controls, the Pearson correlation test was used and genotypes were coded as 0, 1, and 2 to reflect the number of copies of the minor allele.

#### Associations between breast cancer risk and genotypes and haplotypes

Descriptive variables were analyzed by Student *t *test or chi-squared test. All genotype analyses were performed for AA and EA populations separately. A genotypic (co-dominant) model was assumed for SNP effects. When genotype frequencies of the rare homozygote were not more than 5% in both populations, categories were collapsed (homozygote rare and heterozygote) for power considerations. To test whether there was a linear dose effect of the variant alleles (log-additive genetic model for trend test), SNPs were coded as 0, 1, and 2 as described above. Univariate single SNP analysis was performed, and resulting *P *values were plotted after logarithm transformation with accompanying linkage disequilibrium (LD) map by using the snp.plotter R package [29]. Covariates, including age at diagnosis, BMI, European ancestry, family history of breast cancer, and education, were then adjusted in multivariate logistic regression models to derive odds ratios (ORs) and 95% confidence intervals (CIs).

For *VDR *and *CYP24A1*, haplotype structure was determined by using the method of Gabriel and colleagues [30], and each haplotype was tested in the regression model in comparison with all other haplotypes. For both single SNP and haplotype analyses, we controlled the family-wise error rate by using permutation testing (*n *= 10,000) as implemented in PLINK [31].

#### Potential modification of associations by race, menopausal status, and estrogen receptor status

To examine whether the associations of SNPs with breast cancer risk differed between AA and EA women, interaction with race was tested by including a race*SNP term in the logistic regression model without an estimate of ancestry. A similar approach was used to test modification effects by menopausal status.

To test whether selected SNPs contributed to differential risk of ER^- ^breast cancer between AA and EA women, OR of ER^- ^breast cancer by race was first estimated from a base model containing race and other covariates. SNPs that were differentially associated with ER^- ^breast cancer risk by race (*P *for interaction was less than 0.10) were then entered in the base model and subjected to backward selection. A substantial reduction of OR (≥ 10%) for AA versus EA race by adding selected SNPs would indicate that those SNPs explained, in part, the higher risk of ER^- ^breast cancer in AA than in EA women.

## Results

### Descriptive characteristics

Table 1 summarizes the descriptive characteristics of the study population by self-reported race. The majority of the women were pre-menopausal at the time of cancer diagnosis (62%) or enrollment for controls (57%). Overall, AA women had higher BMIs than EA women (31.3 versus 27.2 kg/m^2^) and were less likely to have a college education or beyond (57.5% versus 82.0%), to take hormone replacement therapy after menopause (14.0% versus 24.1%), or to have a family history of breast cancer in first-degree relatives (13.5% versus 22.4%) (all *P *< 0.001). There were no significant case-control differences in AAs or EAs, except that in EA women, cases were less likely than controls to have a college education and more likely to have a positive family history of breast cancer (*P *≤ 0.001).

**Table 1:** Descriptive characteristics of African-American and European-American women by case-control status in the Women's Circle of Health Study

	African-American			European-American		
Characteristics	Case (*n *= 547)	Control (*n *= 461)	*P *value	Case (*n *= 381)	Control (*n *= 382)	*P *value
	Mean (SD)	Mean (SD)		Mean (SD)	Mean (SD)	
Age, years	51.7 (10.0)	49.8 (9.9)	0.003	51.0 (8.4)	50.9 (8.3)	0.82
Body mass index, kg/m^2^	31.2 (6.7)	31.6 (7.8)	0.48	26.8 (5.8)	27.7 (7.1)	0.06
Percentage with European ancestry	0.09 (0.15)	0.10 (0.16)	0.19	0.98 (0.07)	0.99 (0.03)	0.07
	Count (%)	Count (%)		Count (%)	Count (%)	
Menopausal status			0.14			0.17
Pre-menopausal	337 (61.6)	263 (57.0)		235 (61.7)	217 (56.8)	
Post-menopausal	210 (38.4)	198 (43.0)		146 (38.3)	165 (43.2)	
Family history			0.13			0.001
Yes	82 (15.0)	54 (11.7)		104 (27.3)	67 (17.5)	
No	465 (85.0)	407 (88.3)		277 (72.7)	315 (82.5)	
Education			0.06			< 0.001
Less than high school	76 (13.9)	55 (11.9)		9 (2.4)	4 (1.1)	
High school	175 (32.0)	122 (26.5)		80 (21.0)	44 (11.5)	
College or above	296 (54.1)	284 (61.6)		292 (76.6)	334 (87.4)	
Hormone replacement therapy			0.74			0.47
Yes	79 (14.5)	62 (14.2)		96 (25.3)	88 (23.0)	
No	464 (85.5)	397 (85.8)		284 (74.7)	294 (77.0)	

For continuous variables, *P *values were based on the *t *test; for categorical variables, *P *values were based on the chi-squared test. Owing to occasional missing values, the counts of some variables did not add up to the total. SD, standard deviation.

### Serum levels of 25OHD

**Figure 1:**
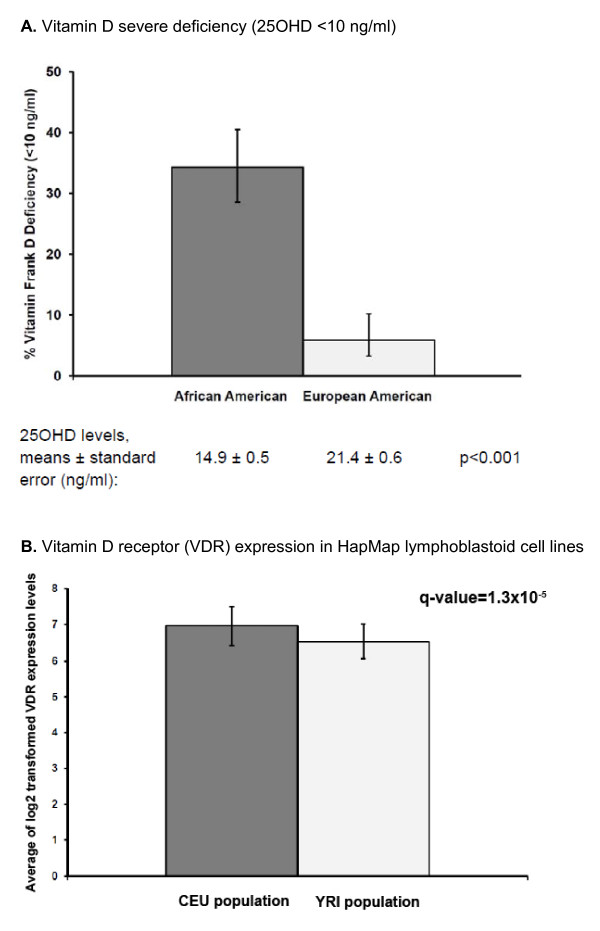
**Serum 25OHD and vitamin D receptor expression in populations of African and European ancestry**. **(a) **The bars indicate the proportion of frank vitamin D deficiency - 25-hydroxyvitamin D (25OHD) of less than 10 ng/mL - and the 95% confidence interval. **(b) **The bars indicate the average of the log_2_-transformed gene expression levels of vitamin D receptor (VDR) in the HapMap lymphoblastoid cell lines from the CEU (Utah residents with ancestry from Northern and Western Europe) population and the YRI (Yoruba in Ibadan) population. The data were obtained from Zhang and colleagues [32].

Among controls, serum levels of 25OHD were lower in AA than EA women (least squares means and standard errors after age, BMI, and season of blood collection were controlled for: 14.9 ± 0.5 versus 21.4 ± 0.6 ng/mL; *P *< 0.001). As shown in Figure 1a, the rate of vitamin D severe deficiency (< 10 ng/mL) was almost sixfold higher in AA than EA women (34.3% versus 5.9%). On the basis of publicly available gene expression data in cultured lymphoblastoid cell lines (LCLs) from the HapMap CEU and YRI populations [32], estimated average expression levels of VDR were significantly lower in LCLs from the African population than in those from the European population (log_2_-transformed level mean ± standard deviation: 6.54 ± 0.47 versus 6.96 ± 0.53; q value after controlling for multiple comparison: 1.30 × 10^-5^) (Figure 1b). We categorized AA women by proportion of African ancestry (< 85%, 85% to 94%, and ≥ 95%) and found that women with the lowest African ancestry had the highest serum 25OHD levels (15.5 ng/mL) but that those with the greatest African ancestry (≥ 95%) had the lowest levels (13.7 ng/mL) (*P *= 0.07). When correlations between SNPs and serum 25OHD levels in AA and EA women were tested, the minor allele of *VDR *SNP rs2239186 was significantly associated with increased levels of 25OHD in AAs. For the AA, AG, and GG genotypes, the means and standard deviations of serum 25OHD were 13.5 ± 6.5, 16.3 ± 8.7, and 21.2 ± 12.2 ng/mL, respectively (*P *= 0.006). However, the differences were not significant in EA women (21.0, 23.8, and 22.1 ng/mL for the AA, AG, and GG genotypes, respectively; *P *= 0.20).

### Associations between genetic variants and breast cancer risk by self-reported race

**Figure 2:**
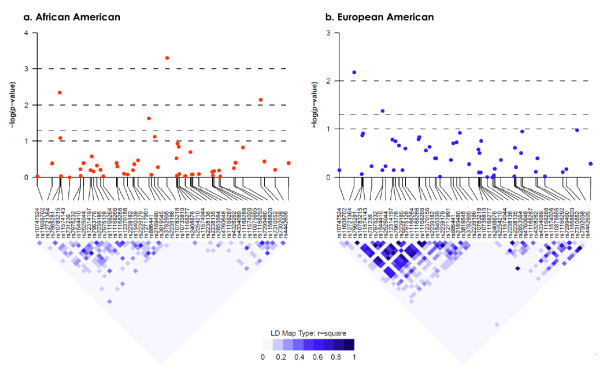
**Logarithm-transformed *P *values derived from univariate single-nucleotide polymorphism analysis of vitamin D receptor (*VDR*)**. *P *values were plotted with accompanying linkage disequilibrium (LD) map for African-American **(a) **and European-American **(b) **separately.

**Figure 3:**
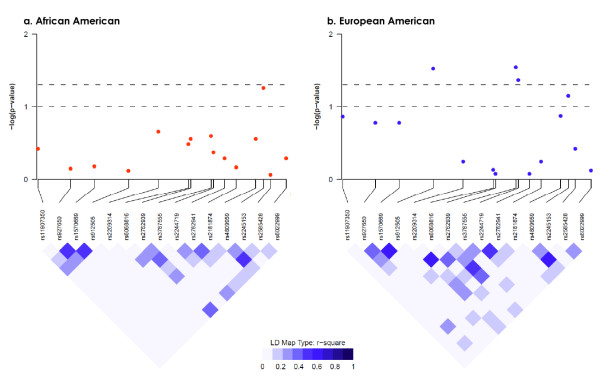
**Logarithm-transformed *P *values derived from univariate single-nucleotide polymorphism analysis of *CYP24A1***. *P *values were plotted with accompanying linkage disequilibrium (LD) map for African-American **(a) **and European-American **(b) **separately.

In addition to circulating 25OHD levels, there were racial differences in genetic variants. Of the 65 SNPs genotyped, 51 (79%) displayed significantly different allele frequencies by self-reported race (*P *< 0.05); 12 of these SNPs were the rare variant in one group (AA or EA) but the common allele in the other group (Table S1 of Additional file 1). LD in *VDR *and *CYP24A1 *also displayed different patterns by race, as shown in Figures 2 and 3. Also shown in the figures are unadjusted *P *values for associations between single SNPs and breast cancer risk (see Table S1 of Additional file 1 for results of all SNPs). In AA women, four SNPs in *VDR *- rs12721364, rs2239186, rs886441, and rs11568820 (Cdx2) - but none in *CYP24A1 *were associated with breast cancer risk at a nominal significance level of 0.05 (Figures 2a and 3a). The association of *VDR *rs2239186 remained significant after correction for multiple testing (*P *= 0.03). In EA women, two SNPs in *VDR *- rs11608702 and rs7975332 (Apa1) - and three SNPs in *CYP24A1 *- rs912505, rs3787555, and rs2244719 - were associated with breast cancer risk (*P *< 0.05) (Figures 2b and 3b) but did not remain significant after multiple comparisons were controlled for (data not shown). There were no associations between the SNP in *CYP27B1 *and breast cancer risk in either EA or AA women.

Table 2 shows ORs and 95% CIs for four SNPs (rs11608702, rs12721364, rs2239186, and rs11568820) in *VDR *and two SNPs (rs912505 and rs3787555) in *CYP24A1 *which had differential associations between AA and EA women (*P *for interaction by race was not more than 0.10) after adjustment for age, proportion of European ancestry, BMI, family history of breast cancer, and education. In AA women, the combined GG and AG genotypes of rs2239186, which remained significant after correction for multiple testing and were also related to increased levels of 25OHD, were associated with an almost 50% reduction of risk of breast cancer in comparison with homozygotes for A alleles (OR = 0.53, 95% CI = 0.35 to 0.79, *P *trend for the G allele = 0.001). Among AA women, a reduced risk associated with *VDR *rs12721364 (OR = 0.53, 95% CI = 0.31 to 0.79, *P *= 0.01) and a marginally increased risk with SNP rs11568820 (Cdx2) (OR for AA genotypes = 1.94, 95% CI = 1.01 to 3.74, *P *= 0.04) were observed.

**Table 2:** Single-nucleotide polymorphisms in *VDR *and *CYP24A1 *and differential associations with breast cancer risk between African-American and European-American women in the Women's Circle of Health Study

			African-American			European-American			
Gene	SNP	Genotype	Cases/Controls	Adjusted OR (95% CI)	*P*_trend_	Cases/Controls	Adjusted OR (95% CI)	*P*_trend_	*P*_interaction_
*VDR*	rs11608702	AA	330/261	1.00	0.37	166/190	1.00	0.02	0.01
		AT	175/175	0.80 (0.61-1.04)		160/159	1.15 (0.84-1.58)		
		TT	37/25	1.13 (0.65-1.95)		55/31	1.88 (1.14-3.09)		
*VDR*	rs12721364	GG	520/420	1.00	0.01	303/298	1.00	0.90	0.02
		GA/AA	24/40	0.53 (0.31-0.90)		78/82	0.98 (0.68-1.41)		
*VDR*	rs2239186	AA	497/393	1.00	0.001^a^	265/256	1.00	0.46	0.01
		AG/GG	47/68	0.53 (0.35-0.79)		115/125	0.85 (0.62-1.17)		
*VDR*	rs11568820 (Cdx2)	GG	18/26	1.00	0.04	234/232	1.00	0.68	0.04
		GA	143/140	1.55 (0.79-3.03)		129/132	0.99 (0.72-1.36)		
		AA	384/295	1.94 (1.01-3.74)		18/18	0.83 (0.39-1.75)		
*CYP24A1*	rs912505	AA	173/139	1.00	0.71	236/216	1.00	0.02	0.05
		AG	268/244	0.89 (0.66-1.19)		132/139	0.80 (0.59-1.10)		
		GG	104/77	1.14 (0.78-1.66)		13/27	0.36 (0.17-0.76)		
*CYP24A1*	rs3787555	CC	379/331	1.00	0.14	206/183	1.00	0.03	0.02
		CA	154/123	1.17 (0.88-1.56)		149/155	0.81 (0.59-1.10)		
		AA	14/7	1.88 (0.73-4.83)		25/41	0.50 (0.28-0.89)		

Odds ratio (OR) and 95% confidence interval (CI) are adjusted for covariates, including age, proportion of European ancestry, body mass index, family history of breast cancer, and education. *P*_trend _was for genetic dose response by coding genotypes as 0, 1, and 2 on the basis of the number of variant alleles. *P*_interaction _was for the differences in ORs between African-American and European-American women, and a *P*_interaction _of less than 0.10 was deemed significant. ^a^Single-nucleotide polymorphism (SNP) rs2239186 in *VDR *remained significant after correction for multiple comparison by permutation.

Among EA women, the *VDR *'at-risk' G allele for rs2239186 was more common in EA women but was not associated with breast cancer risk (OR = 0.85, 95% CI = 0.62 to 1.17), nor were *VDR *rs12721364 SNPs. There were increases in risk by the *VDR *SNP rs11608702 and significant decreases in risk by two *CYP24A1 *variants: rs912505 and rs3787555; however, these did not remain significant after correction for multiple testing.

Results from haplotype analysis were consistent with those from single SNP analysis for *VDR *rs2239186. Among AA women, a G-G-G haplotype consisting of this SNP and two neighboring variants was associated with a decreased risk of breast cancer after adjustment for multiple testing (OR = 0.55, 95% CI = 0.38 to 0.81, *P *= 0.04) (Table S2 of Additional file 1). Among EA women, similar results were found for haplotypes containing rs11608702 in *VDR *and haplotypes containing rs3787555 in *CYP24A1*. The commonly studied haplotype in the 3' untranslated region of *VDR *consisting of Taq1, Apa1, and Bsm1 was not associated with breast cancer risk in AA women, but a modest decreased risk was observed in EA women and significance was marginal (OR = 0.82, 95% CI = 0.67 to 1.02).

### Estrogen receptor-negative breast cancer and *CYP24A1 *variants

Stratification by ER status revealed associations that were not observed in the overall analysis, and the majority of findings were observed only for ER^- ^breast cancer (Tables S3 and S4 of Additional file 1). Although *VDR *rs10783218 was marginally associated with a twofold increased risk of ER^+ ^breast cancer among EA women and *VDR *rs3819545 was associated with a decreased risk of ER^- ^breast cancer, several SNPs in *CYP24A1 *were highly significantly associated with risk of ER^- ^breast cancer. Importantly, results differed markedly between AA and EA women (*P *for interaction was not more than 0.10) (Table 3). For example, *CYP24A1 *rs27622941 was associated with a more than twofold increased risk of ER^- ^breast cancer among AA women (OR = 2.62, 95% CI = 1.38 to 4.98) and there were no associations in EAs (OR = 0.78, 95% CI = 0.34 to 1.78). Conversely, *CYP24A1 *rs2209314 was associated with an almost threefold decreased risk of ER^- ^breast cancer in EA women (OR = 0.38, 95% CI = 0.20 to 0.73) and there were no associations in AA women (OR = 1.34, 95% CI = 0.74 to 2.40).

**Table 3:** Single-nucleotide polymorphisms in *VDR *and *CYP24A1 *and differential association with estrogen receptor-specific breast cancer risk among African-American and European-American women

			African-American			European-American			
Gene	SNP	Genotype	Cases/Controls	OR (95% CI)	*P*_trend_	Cases/Controls	OR (95% CI)	*P*_trend_	*P*_interaction_
*VDR*	rs10783218	GG	178/304	1.00	0.82	194/352	1.00	0.04	0.04
		GA/AA	85/150	0.96 (0.69-1.34)		20/17	2.05 (1.02-4.12)		
*VDR*	rs3819545	AA	71/242	1.00	0.04	22/162	1.00	0.38	0.04
		AG	45/168	0.91 (0.59-1.40)		26/156	1.22 (0.66-2.26)		
		GG	3/43	0.23 (0.07-0.77)		11/52	1.40 (0.62-3.15)		
*CYP24A1*	rs927650	GG	66/258	1.00	0.62	10/116	1.00	0.003	0.10
		GA	47/170	1.09 (0.71-1.68)		29/186	1.76 (0.81-3.78)		
		AA	8/27	1.18 (0.50-2.79)		20/68	3.46 (1.50-7.96)		
*CYP24A1*	rs1570669	GG	15/81	1.00	0.69	32/158	1.00	0.03	0.05
		GA	69/219	1.77 (0.94-3.31)		24/162	0.71 (0.40-1.27)		
		AA	37/154	1.36 (0.69-2.67)		3/50	0.28 (0.08-0.97)		
*CYP24A1*	rs2209314	AA	102/393	1.00	0.33	46/208	1.00	0.004	0.003
		AG/GG	19/61	1.34 (0.74-2.40)		13/162	0.38 (0.20-0.73)		
*CYP24A1*	rs3787555	CC	79/326	1.00	0.02	25/175	1.00	0.91	0.09
		CA	37/122	1.42 (0.90-2.24)		29/155	1.25 (0.69-2.24)		
		AA	5/7	3.79 (1.11-12.91)		5/38	0.83 (0.30-2.35)		
*CYP24A1*	rs2762941	AA	16/120	1.00	0.004	24/132	1.00	0.54	0.05
		AG	61/212	1.97 (1.07-3.61)		26/168	0.89 (0.49-1.65)		
		GG	44/122	2.62 (1.38-4.98)		9/68	0.78 (0.34-1.78)		
*CYP24A1*	rs4809959	GG	32/133	1.00	0.99	14/131	1.00	0.01	0.07
		GA	64/223	1.13 (0.70-1.83)		26/172	1.43 (0.71-2.88)		
		AA	25/98	0.98 (0.54-1.78)		19/66	2.71 (1.25-5.86)		
*CYP24A1*	rs2585428	GG	27/109	1.00	0.65	26/90	1.00	0.006	0.02
		GA	66/246	1.12 (0.67-1.87)		22/175	0.46 (0.24-0.87)		
		AA	28/100	1.15 (0.63-2.10)		11/105	0.36 (0.17-0.79)		

Odds ratio (OR) and 95% confidence interval (CI) are adjusted for covariates, including age, proportion of European ancestry, body mass index, family history of breast cancer, and education. *P*_trend _was for genetic dose response by coding genotypes as 0, 1, and 2 on the basis of the number of variant alleles. *P*_interaction _was for the differences in ORs between African-American and European-American women, and a *P*_interaction _of less than 0.10 was deemed significant. SNP, single-nucleotide polymorphism.

To determine whether these SNPs contributed to the observed higher risk of ER^- ^breast cancer in AA women in comparison with EA women, a base model containing self-reported race and other covariates was developed (Table 4). The base model showed an increased ER^- ^cancer risk associated with AA race (OR = 1.53, 95% CI = 1.06 to 2.22). The eight SNPs that showed significant interactions with race were tested in the base model. After backward selection, the two *CYP24A1 *SNPs shown above, rs2209314 and rs2762941, remained significant in the final model, reducing the risk associated with AA race by 22% and rendering it non-significant (OR = 1.20, 95% CI = 0.80 to 1.79).

**Table 4:** Changes in risk of estrogen receptor-negative breast cancer by race with inclusion of single-nucleotide polymorphisms in *CYP24A1*

Model	Variable	Adjusted OR (95% CI)	*P *value
Base model	Race (AA versus EA)	1.53 (1.06-2.22)	0.02

Base model + SNPs	Race (AA versus EA)	1.20 (0.80-1.79)	0.38

	rs2209314 (AG/GG versus AA)	0.57 (0.36-0.89)	0.01

	rs2762941 (AG versus AA)	1.47 (0.96-2.25)	0.04

	rs2762941 (GG versus AA)	1.88 (1.15-3.06)	

Covariates included in the base model were age at diagnosis, body mass index, family history of breast cancer, education, and race. Odds ratio (OR) and 95% confidence interval (CI) for race after adjustment for other covariates are shown. Based on this model, seven single-nucleotide polymorphisms (SNPs) in *CYP24A1 *(rs927650, rs1570669, rs2209314, rs3787555, rs2762941, rs4809959, and rs2585428) and one SNP in *VDR *(rs3819545) that were associated with estrogen receptor-negative (ER^-^) breast cancer risk in either African-American (AA) or European-American (EA) women were entered and backward-selected. Two SNPs, rs2209314 and rs2762941, remained in the final model with a *P *value of less than 0.05. ORs and 95% CIs for race and the two SNPs are shown.

Lastly, there were significant interactions for two SNPs in *VDR *with menopausal status (Table S5 of Additional file 1). The increased risk associated with rs886441 in AA women was restricted to pre-menopausal women (OR = 2.27, 95% CI = 1.32 to 3.90), and the increased risk associated with rs7975232 (Apa1) in EA women was restricted to post-menopausal women (OR = 2.24, 95% CI = 1.19 to 4.21).

## Discussion

In this study, we found that relationships between breast cancer risk and variants in genes associated with vitamin D activity and metabolism, *VDR *and *CYP24A1*, differed depending upon self-reported race and that associations were most notable for risk of ER^- ^breast cancer in both AA and EA women. Importantly, we found that rs2209314 and rs2762941 in *CYP24A1 *contributed significantly to the higher risk of ER^- ^breast cancer in AA than EA women. Among controls in the WCHS, serum levels of 25OHD were notably lower in AA women than EA women, the lowest levels were among women with the greatest African ancestry estimated by AIMs, and VDR expression levels, as estimated from published data on cultured lymphoblastoid cells [32], were also lower in AA women. In a previous study among EA women, we found that low 25OHD levels were associated with increased risk of ER^- ^breast cancer, both in comparison with controls and with women with ER^+ ^breast cancer [22]. Given all of these data, it is possible that low 25OHD levels in AA women, coupled with unique 'at-risk' genetic variants, contribute, in part, to the higher prevalence of ER^- ^breast cancer among AA women. If these potential associations were to be consistently observed in future studies, our results would support a public health effort for vitamin D supplementation to reduce risk of aggressive breast cancer among AA women.

The finding of an inverse association between African ancestry estimated by AIMs and blood 25OHD levels is consistent with a recent community cohort study of AA men and women [33]. Our findings of extensive racial differences in allele frequencies and LD patterns for SNPs in *VDR *and *CYP24A1 *are also consistent with those from an earlier study [12]. Previous studies on *VDR *polymorphisms and breast cancer risk have focused on only a few SNPs. However, we did not find any relationship with Fok1, Bsm1, or Taq1 or a three-SNP haplotype consisting of Bsm1, Apa1, and Taq1 in either AA or EA women. The variant homozygote of Apa1 was associated with increased risk of breast cancer in EA women, but the effect was limited to post-menopausal women. Increased risk of breast cancer was also reported in a previous study for Apa1 [34]; however, results in the literature are conflicting [35,36]. The G allele of Cdx2 was associated with lower risk of breast cancer in AA women in our study and this finding was in contrast to the speculated functional alteration that the variant G allele resulted in lower binding of the Cdx2 protein and thus lower transcriptional activity of VDR [37,38]. Similar to a study among women in Germany [39], our study found no association of Cdx2 with breast cancer risk in EA women.

To date, three studies have examined selected SNPs in *VDR *with breast cancer risk in both AA and EA women. Two of them did not find associations for Fok1, Bsm1, Bgl1 (rs739837), or the 3' untranslated region poly(A) microsatellite in either AA or EA women [40,41], and a third study found increased risk by Bsm1 variant in EA but not AA women [42]. However, none of the above studies examined the associations by ER status or used the systematic approach we employed to capture variation across the genes.

We found that four SNPs in *VDR *and two SNPs in *CYP24A1 *had differential associations with breast cancer by race (*P *for interaction was not more than 0.10). The fact that the associations were not consistent in AA and EA populations corroborates the differences in blood levels of vitamin D and frequency and LD pattern of vitamin D-related genetic variants, implying that the race-specific associations might be the result of gene-environment interactions. In further analyses stratified by ER status, one SNP in *VDR *and seven SNPs in *CYP24A1 *were specifically associated with ER^- ^but not ER^+ ^cancer risk, and the associations differed between AA and EA women. Controlling for the two SNPs in *CYP24A1 *in a multivariate model substantially reduced the increased ER^- ^breast cancer risk associated with AA race and made the association no longer significant. This provides the first evidence supporting the contribution of vitamin D-related genetic variants to higher risk of more aggressive breast cancer in AA women.

We found significant associations between breast cancer risk and a number of tag SNPs in *VDR *without previously known functionality. However, in our analyses, SNP rs2239186 was associated with increased serum levels of 25OHD in AA women without breast cancer. This SNP and a haplotype containing it were also significantly associated with reduced breast cancer risk in AA women, irrespectively of ER status. This SNP resides in an intronic region and thus is unlikely to be the causal variant. However, it may be a marker for a causal SNP outside of the *VDR *gene. This SNP has not been implicated in other breast cancer studies but has been shown to be associated with reduced risk of colorectal cancer in individuals with low vitamin D intake [43] and was also implicated in type I diabetes [44]. This SNP may warrant future replication and fine-mapping studies.

The two SNPs, rs2209314 and rs2762941, in *CYP24A1 *shown to be associated with racial differences in ER^- ^breast cancer risk are intronic. Although these SNPs have not been implicated previously in breast cancer, elevated expression of CYP24A1 was found in breast cancer tissues [45], indicating a potential role in breast cancer etiology. We did not observe associations of these two SNPs in *CYP24A1 *with serum 25OHD levels in either AA or EA populations, indicating that these two SNPs themselves or linked causal variants may affect ER^- ^breast cancer not through altering circulating 25OHD levels but availability of vitamin D in local mammary tissues.

One limitation of our study is the lack of validation for the significant findings. The number of patients with breast cancer and controls was relatively limited, especially after stratification by race and ER status. None of the associations with SNPs, except for rs2239186, remained significant after correction for multiple comparisons. We thus could not exclude the possibility of false-positive findings in our data. However, the fact that rs2239186 was associated with higher serum 25OHD levels in AA women as well as reduced breast cancer risk in this population is biologically coherent and reduces the likelihood of spurious findings for this *VDR *SNP. Another limitation is that only three genes in vitamin D-related pathways were included in this study. Although *VDR*, *CYP27B1*, and *CYP24A1 *are the three key genes in this pathway, genes encoding for some other vitamin D metabolizing enzymes, particularly *GC *encoding for vitamin D binding protein (which has been related to circulating vitamin D levels), may also be related to breast cancer risk and warrant further studies. AA women are more likely to develop breast cancer at a younger age than EAs; we enrolled all eligible AA women but randomly selected eligible EA women, frequency-matching by 5-year age categories. We also initially limited eligibility to women 65 years or younger because of low participation of older women without breast cancer to case-control studies. Thus, the overall study population is younger than that of some other studies. Although we found no evidence of modification effects by menopausal status for any but two SNPs (Table S5 of Additional file 1), the high proportion of pre-menopausal women in this study needs to be considered in relation to generalizability.

## Conclusions

We found notable differences in blood levels of 25OHD and genetic variants in *VDR *and *CYP24A1 *between AA and EA women. Specifically, we found race-specific associations with breast cancer risk and these associations may be due to distinct genetic background and differences in 25OHD levels between the two populations. Our study provides evidence that variants in vitamin D-related genes may contribute to higher risk of ER^- ^breast cancer in AA than EA women. Future studies are warranted to validate our findings and to investigate whether pre-diagnostic blood levels of 25OHD may also be related to racial differences in risk of ER^- ^breast cancer.

## Abbreviations

25OHD: 25-hydroxyvitamin D; AA: African-American; AIM: ancestry informative marker; BMI: body mass index; CEU: Utah residents with ancestry from Northern and Western Europe; CI: confidence interval; EA: European-American; ER: estrogen receptor; LCL: lymphoblastoid cell line; LD: linkage disequilibrium; OR: odds ratio; PR: progesterone receptor; SNP: single-nucleotide polymorphism; UV: ultraviolet; VDR: vitamin D receptor; WCHS: Women's Circle of Health Study; YRI: Yoruba in Ibadan.

## Competing interests

The authors declare that they have no competing interests.

## Authors' contributions

SY helped to develop the hypothesis, design the study, and perform the data analysis and interpretation. CBA helped to develop the hypothesis, design the study, provide the data and biospecimen from the WCHS, perform the data analysis and interpretation, and provide financial support for the study. GZ helped to provide the data and biospecimen from the WCHS and to perform the data analysis and interpretation. DHB, LJ, MR, GC, WD, KSP, and EVB helped to provide the data and biospecimen from the WCHS. HH provided pathological data of tumor samples. C-CH, LES, LT, CSJ, DLT, SEM and FA helped to perform the data analysis and interpretation. HZ helped to provide financial support for the study. All authors read and approved the final draft of the manuscript.

## Supplementary Material

Additional file 1 **Supplementary Tables S1-S5**. The file contains the following five supplementary tables. Table S1. Breast cancer risk associated with SNPs in VDR, CYP27B1, and CYP24A1 in African American and European American women. Table S2. Haplotypes of VDR and CYP24A1 in significant association with breast cancer risk in African American and European American women. Table S3. Risk of estrogen receptor positive breast cancer associated with SNPs in VDR, CYP27B1, and CYP24A1 in African Americana and European American women. Table S4. Risk of estrogen receptor negative breast cancer associated with SNPs in VDR, CYP27B1, and CYP24A1 in African Americana and European American women. Table S5. SNPs in VDR that show differential associations with breast cancer stratified by menopausal status in African American and European American women. Click here for file
