# Supplementary material for: Variants in the vitamin D pathway, serum levels of vitamin D, and estrogen receptor negative breast cancer among African-American women: a case-control study
Source: Breast Cancer Res. 2012 Apr 4;14(2):R58. doi: 10.1186/bcr3162 (PMC3446393; doi:10.1186/bcr3162)
Supplement: Additional file 1 — Supplementary Tables S1-S5. The file contains the following five supplementary tables. Table S1. Breast cancer risk associated with SNPs in VDR, CYP27B1, and CYP24A1 in African American and European American women. Table S2. Haplotypes of VDR and CYP24A1 in significant association with breast cancer risk in African American and European American women. Table S3. Risk of estrogen receptor positive breast cancer associated with SNPs in VDR, CYP27B1, and CYP24A1 in African Americana and European American women. Table S4. Risk of estrogen receptor negative breast cancer associated with SNPs in VDR, CYP27B1, and CYP24A1 in African Americana and European American women. Table S5. SNPs in VDR that show differential associations with breast cancer stratified by menopausal status in African American and European American women. [file bcr3162-S1.DOC]

**Supplementary Table S1. Breast cancer risk associated with SNPs in *VDR*, *CYP27B1*, and *CYP24A1* in African American and European American women**

| **Gene** | **SNP** | **Chr.** | **Coordinate** | **Minor Allele** | **African American** | | | |  | **European American** | | | | **Pinteraction** |
| --- | --- | --- | --- | --- | --- | --- | --- | --- | --- | --- | --- | --- | --- | --- |
|  |  |  |  |  | **MAF** | **OR (95% CI) per variant allele** | **Praw** | **Ppermutation** |  | **MAF** | **OR (95% CI) per variant allele** | **Praw** | **Ppermutation** |  |
| VDR | rs10747524 | 12 | 46509741 | G | 0.40 | 0.99 (0.83-1.19) | 0.94 | 1.00 |  | 0.44 | 1.04 (0.84-1.29) | 0.71 | 1.00 | 0.74 |
| VDR | rs11608702 | 12 | 46515035 | T | 0.24 | 0.92 (0.75-1.13) | 0.42 | 1.00 |  | 0.32 | 1.35 (1.09-1.67) | 0.007 | 0.25 | 0.01 |
| VDR | rs12721364 | 12 | 46517697 | A | 0.03 | 0.48 (0.29-0.81) | 0.005 | 0.22 |  | 0.11 | 0.97 (0.71-1.33) | 0.86 | 1.00 | 0.02 |
| VDR | rs7965281 | 12 | 46517877 | G | 0.40 | 0.85 (0.71-1.02) | 0.08 | 0.98 |  | 0.51 | 0.86 (0.70-1.05) | 0.14 | 1.00 | 0.97 |
| VDR | rs10783215 | 12 | 46518165 | G | 0.39 | 0.99 (0.83-1.19) | 0.91 | 1.00 |  | 0.47 | 1.17 (0.96-1.43) | 0.12 | 0.99 | 0.22 |
| VDR | rs11574143 | 12 | 46521184 | A | 0.08 | 1.00 (0.72-1.39) | 0.99 | 1.00 |  | 0.12 | 0.92 (0.67-1.26) | 0.59 | 1.00 | 0.70 |
| VDR | rs731236 | 12 | 46525024 | G | 0.30 | 0.95 (0.78-1.15) | 0.59 | 1.00 |  | 0.38 | 0.96 (0.78-1.18) | 0.71 | 1.00 | 0.93 |
| VDR | rs7975232 | 12 | 46525104 | C | 0.33 | 1.01 (0.84-1.22) | 0.90 | 1.00 |  | 0.40 | 1.24 (1.01-1.52) | 0.04 | 0.81 | 0.16 |
| VDR | rs1544410 | 12 | 46526102 | A | 0.32 | 0.92 (0.76-1.11) | 0.40 | 1.00 |  | 0.39 | 0.95 (0.77-1.16) | 0.59 | 1.00 | 0.87 |
| VDR | rs2525044 | 12 | 46528523 | A | 0.19 | 1.06 (0.85-1.32) | 0.63 | 1.00 |  | 0.42 | 1.16 (0.94-1.42) | 0.17 | 1.00 | 0.57 |
| VDR | rs12314197 | 12 | 46528989 | G | 0.23 | 1.13 (0.91-1.39) | 0.27 | 1.00 |  | 0.00 | 1.34 (0.30-5.99) | 0.70 | 1.00 | 0.83 |
| VDR | rs7963776 | 12 | 46529644 | G | 0.39 | 1.04 (0.87-1.24) | 0.68 | 1.00 |  | 0.45 | 1.15 (0.94-1.41) | 0.18 | 1.00 | 0.47 |
| VDR | rs2239185 | 12 | 46530826 | G | 0.41 | 1.07 (0.89-1.28) | 0.47 | 1.00 |  | 0.45 | 1.14 (0.93-1.39) | 0.22 | 1.00 | 0.66 |
| VDR | rs7975128 | 12 | 46532095 | A | 0.29 | 0.95 (0.78-1.16) | 0.62 | 1.00 |  | 0.39 | 0.96 (0.78-1.18) | 0.70 | 1.00 | 0.95 |
| VDR | rs11168264 | 12 | 46533130 | G | 0.24 | 1.01 (0.82-1.24) | 0.94 | 1.00 |  | 0.005 | 2.51 (0.49-12.98) | 0.26 | 1.00 | 0.26 |
| VDR | rs11168266 | 12 | 46537800 | G | 0.40 | 1.08 (0.90-1.29) | 0.41 | 1.00 |  | 0.44 | 1.15 (0.94-1.41) | 0.17 | 1.00 | 0.63 |
| VDR | rs11168268 | 12 | 46538079 | G | 0.35 | 1.07 (0.89-1.28) | 0.50 | 1.00 |  | 0.43 | 1.16 (0.95-1.42) | 0.15 | 1.00 | 0.54 |
| VDR | rs12370156 | 12 | 46540400 | G | 0.49 | 1.02 (0.86-1.22) | 0.80 | 1.00 |  | 0.48 | 1.12 (0.91-1.37) | 0.28 | 1.00 | 0.52 |
| VDR | rs2239182 | 12 | 46541678 | A | 0.42 | 1.02 (0.85-1.22) | 0.83 | 1.00 |  | 0.47 | 1.13 (0.92-1.38) | 0.24 | 1.00 | 0.46 |
| VDR | rs1540339 | 12 | 46543593 | A | 0.23 | 0.95 (0.77-1.17) | 0.63 | 1.00 |  | 0.33 | 1.10 (0.88-1.38) | 0.41 | 1.00 | 0.35 |
| VDR | rs2239179 | 12 | 46544033 | G | 0.33 | 0.93 (0.77-1.12) | 0.43 | 1.00 |  | 0.44 | 0.92 (0.75-1.12) | 0.41 | 1.00 | 0.94 |
| VDR | rs12717991 | 12 | 46545393 | A | 0.32 | 0.91 (0.76-1.10) | 0.35 | 1.00 |  | 0.34 | 1.00 (0.81-1.24) | 0.97 | 1.00 | 0.52 |
| VDR | rs886441 | 12 | 46549231 | G | 0.39 | 1.23 (1.03-1.48) | 0.02 | 0.68 |  | 0.22 | 1.10 (0.86-1.40) | 0.44 | 1.00 | 0.46 |
| VDR | rs2189480 | 12 | 46550095 | A | 0.36 | 1.02 (0.85-1.22) | 0.85 | 1.00 |  | 0.34 | 1.15 (0.93-1.42) | 0.20 | 1.00 | 0.40 |
| VDR | rs3819545 | 12 | 46551273 | G | 0.26 | 0.83 (0.68-1.02) | 0.08 | 0.97 |  | 0.37 | 1.15 (0.93-1.42) | 0.19 | 1.00 | 0.03 |
| VDR | rs3782905 | 12 | 46552434 | C | 0.22 | 0.93 (0.76-1.15) | 0.53 | 1.00 |  | 0.33 | 0.84 (0.68-1.05) | 0.12 | 0.99 | 0.51 |
| VDR | rs2239186 | 12 | 46555677 | G | 0.06 | 0.52 (0.35-0.75) | 0.0005 | 0.03 |  | 0.17 | 0.92 (0.70-1.20) | 0.54 | 1.00 | 0.01 |
| VDR | rs10783218 | 12 | 46559010 | A | 0.19 | 1.12 (0.90-1.41) | 0.31 | 1.00 |  | 0.03 | 1.43 (0.76-2.68) | 0.27 | 1.00 | 0.49 |
| VDR | rs2228570 | 12 | 46559162 | A | 0.23 | 0.99 (0.80-1.22) | 0.89 | 1.00 |  | 0.37 | 0.97 (0.79-1.20) | 0.79 | 1.00 | 0.93 |
| VDR | rs11168277 | 12 | 46559327 | G | 0.14 | 1.23 (0.95-1.58) | 0.12 | 1.00 |  | 0.001 | NA | 0.32 | 1.00 | 0.37 |
| VDR | rs2408876 | 12 | 46559832 | G | 0.49 | 1.14 (0.96-1.36) | 0.15 | 1.00 |  | 0.41 | 0.97 (0.79-1.19) | 0.79 | 1.00 | 0.25 |
| VDR | rs2254210 | 12 | 46559981 | A | 0.33 | 0.98 (0.81-1.18) | 0.81 | 1.00 |  | 0.33 | 1.16 (0.94-1.44) | 0.18 | 1.00 | 0.24 |
| VDR | rs11574044 | 12 | 46562101 | C | 0.25 | 0.99 (0.81-1.21) | 0.92 | 1.00 |  | 0.16 | 1.00 (0.76-1.32) | 0.98 | 1.00 | 0.93 |
| VDR | rs2238136 | 12 | 46563980 | A | 0.08 | 0.81 (0.59-1.12) | 0.21 | 1.00 |  | 0.26 | 1.00 (0.80-1.26) | 1.00 | 1.00 | 0.30 |
| VDR | rs2238135 | 12 | 46564457 | C | 0.32 | 0.98 (0.81-1.18) | 0.83 | 1.00 |  | 0.26 | 0.99 (0.78-1.24) | 0.91 | 1.00 | 0.97 |
| VDR | rs2853564 | 12 | 46564754 | G | 0.12 | 1.03 (0.79-1.35) | 0.82 | 1.00 |  | 0.38 | 0.95 (0.78-1.18) | 0.66 | 1.00 | 0.66 |
| VDR | rs4760648 | 12 | 46566932 | G | 0.46 | 1.02 (0.86-1.22) | 0.81 | 1.00 |  | 0.56 | 0.92 (0.75-1.13) | 0.44 | 1.00 | 0.46 |
| VDR | rs11168287 | 12 | 46571681 | G | 0.26 | 1.04 (0.85-1.27) | 0.70 | 1.00 |  | 0.52 | 0.99 (0.81-1.22) | 0.96 | 1.00 | 0.76 |
| VDR | rs4328262 | 12 | 46571915 | C | 0.32 | 1.02 (0.84-1.23) | 0.87 | 1.00 |  | 0.43 | 1.13 (0.92-1.38) | 0.25 | 1.00 | 0.46 |
| VDR | rs4334089 | 12 | 46572282 | G | 0.38 | 0.96 (0.80-1.15) | 0.68 | 1.00 |  | 0.76 | 0.94 (0.74-1.19) | 0.61 | 1.00 | 0.88 |
| VDR | rs11168288 | 12 | 46574053 | A | 0.13 | 1.07 (0.82-1.38) | 0.64 | 1.00 |  | 0.001 | NA | 0.32 | 1.00 | 0.33 |
| VDR | rs11574026 | 12 | 46574513 | A | 0.02 | 0.98 (0.53-1.79) | 0.94 | 1.00 |  | 0.12 | 0.77 (0.56-1.06) | 0.11 | 0.99 | 0.51 |
| VDR | rs10875695 | 12 | 46579304 | A | 0.50 | 1.05 (0.88-1.26) | 0.56 | 1.00 |  | 0.76 | 1.04 (0.82-1.31) | 0.77 | 1.00 | 0.91 |
| VDR | rs11168292 | 12 | 46579872 | C | 0.15 | 0.90 (0.70-1.15) | 0.40 | 1.00 |  | 0.33 | 0.91 (0.74-1.13) | 0.41 | 1.00 | 0.92 |
| VDR | rs7299460 | 12 | 46582535 | G | 0.24 | 0.86 (0.70-1.06) | 0.15 | 1.00 |  | 0.70 | 0.99 (0.80-1.24) | 0.95 | 1.00 | 0.35 |
| VDR | rs11568820 | 12 | 46588812 | G | 0.18 | 0.73 (0.58-0.92) | 0.007 | 0.32 |  | 0.78 | 1.04 (0.81-1.32) | 0.78 | 1.00 | 0.04 |
| VDR | rs7310552 | 12 | 46590126 | G | 0.08 | 0.86 (0.62-1.19) | 0.37 | 1.00 |  | 0.40 | 0.96 (0.78-1.18) | 0.68 | 1.00 | 0.59 |
| VDR | rs7302038 | 12 | 46593908 | A | 0.46 | 1.05 (0.88-1.25) | 0.62 | 1.00 |  | 0.01 | 2.53 (0.79-8.09) | 0.11 | 0.98 | 0.13 |
| VDR | rs4442605 | 12 | 46598706 | A | 0.31 | 1.08 (0.90-1.31) | 0.41 | 1.00 |  | 0.21 | 0.92 (0.72-1.18) | 0.53 | 1.00 | 0.31 |
| CYP27B1 | rs4646536 | 12 | 56444255 | G | 0.26 | 1.17 (0.95-1.43) | 0.14 | 1.00 |  | 0.29 | 0.93 (0.74-1.16) | 0.52 | 1.00 | 0.14 |
| CYP24A1 | rs11907350 | 20 | 52203846 | A | 0.18 | 1.11 (0.88-1.40) | 0.38 | 1.00 |  | 0.04 | 0.67 (0.39-1.14) | 0.14 | 1.00 | 0.09 |
| CYP24A1 | rs927650 | 20 | 52206148 | A | 0.25 | 1.04 (0.85-1.27) | 0.71 | 1.00 |  | 0.46 | 1.15 (0.94-1.41) | 0.17 | 1.00 | 0.48 |
| CYP24A1 | rs1570669 | 20 | 52207834 | A | 0.41 | 0.96 (0.80-1.15) | 0.66 | 1.00 |  | 0.67 | 1.16 (0.94-1.44) | 0.17 | 1.00 | 0.18 |
| CYP24A1 | rs912505 | 20 | 52210248 | G | 0.43 | 1.03 (0.86-1.23) | 0.76 | 1.00 |  | 0.23 | 0.77 (0.60-0.97) | 0.03 | 0.69 | 0.05 |
| CYP24A1 | rs2209314 | 20 | 52212368 | G | 0.08 | 1.23 (0.88-1.72) | 0.22 | 1.00 |  | 0.25 | 0.94 (0.74-1.18) | 0.57 | 1.00 | 0.19 |
| CYP24A1 | rs6068816 | 20 | 52214498 | A | 0.05 | 1.23 (0.81-1.87) | 0.33 | 1.00 |  | 0.12 | 1.05 (0.78-1.43) | 0.74 | 1.00 | 0.56 |
| CYP24A1 | rs2762939 | 20 | 52214658 | C | 0.44 | 1.10 (0.92-1.32) | 0.28 | 1.00 |  | 0.74 | 0.98 (0.78-1.23) | 0.83 | 1.00 | 0.41 |
| CYP24A1 | rs3787555 | 20 | 52216098 | A | 0.16 | 1.15 (0.90-1.47) | 0.25 | 1.00 |  | 0.29 | 0.78 (0.62-0.97) | 0.03 | 0.68 | 0.02 |
| CYP24A1 | rs2244719 | 20 | 52216265 | G | 0.25 | 1.09 (0.89-1.33) | 0.43 | 1.00 |  | 0.42 | 1.24 (1.01-1.52) | 0.04 | 0.82 | 0.38 |
| CYP24A1 | rs2762941 | 20 | 52217059 | G | 0.49 | 0.94 (0.79-1.13) | 0.52 | 1.00 |  | 0.59 | 1.02 (0.83-1.25) | 0.83 | 1.00 | 0.56 |
| CYP24A1 | rs2181874 | 20 | 52217885 | A | 0.40 | 0.96 (0.81-1.15) | 0.68 | 1.00 |  | 0.26 | 1.07 (0.85-1.34) | 0.58 | 1.00 | 0.49 |
| CYP24A1 | rs4809959 | 20 | 52219266 | A | 0.48 | 1.10 (0.92-1.31) | 0.28 | 1.00 |  | 0.43 | 1.17 (0.95-1.43) | 0.13 | 0.99 | 0.67 |
| CYP24A1 | rs2245153 | 20 | 52219813 | G | 0.34 | 0.83 (0.68-1.01) | 0.06 | 0.92 |  | 0.24 | 0.80 (0.63-1.02) | 0.07 | 0.94 | 0.83 |
| CYP24A1 | rs2585428 | 20 | 52220304 | A | 0.49 | 0.98 (0.83-1.17) | 0.86 | 1.00 |  | 0.51 | 0.91 (0.75-1.12) | 0.38 | 1.00 | 0.59 |
| CYP24A1 | rs6022999 | 20 | 52221420 | A | 0.36 | 1.06 (0.88-1.28) | 0.52 | 1.00 |  | 0.72 | 1.04 (0.83-1.30) | 0.76 | 1.00 | 0.86 |

*Footnote*: The odds ratio (OR) and 95% confidence interval (CI) are derived from additive genetic model. Ppermuation was used to control for multiple comparison based on 10,000 permutations. Pinteraction was for the differences in odds ratios between African American and European American women. *Abbreviation*: MAF, minor allele frequency. Abbreviation: MAF, minor allele frequency.

**Supplementary Table S2. Haplotypes of *VDR* and *CYP24A1* in significant association with breast cancer risk in African American and European American women**

| **Race** | **Gene** | **Haplotype SNPs** | **Risk haplotype** | **Haplotype frequency in cases** | **Haplotype frequency in controls** | **OR (95% CI) per risk haplotype** | **Punadjusted** | **Ppermutation** |
| --- | --- | --- | --- | --- | --- | --- | --- | --- |
| AA | VDR | rs3819545-rs3782905-rs2239186 | G-G-G | 0.04 | 0.08 | 0.55 (0.38-0.81) | 0.003 | 0.04 |
| AA | VDR | rs731236-rs7975232-rs1544410 (Taq1-Apa1-Bsm1) | G-A-G | 0.07 | 0.06 | 1.19 (0.80-1.76) | 0.82 | 1.00 |
| EA | VDR | rs11608702-rs12721364 | T-G | 0.24 | 0.18 | 1.44 (1.12-1.85) | 0.008 | 0.08 |
| EA | VDR | rs731236-rs7975232-rs1544410 (Taq1-Apa1-Bsm1) | A-A-G | 0.19 | 0.22 | 0.82 (0.67-1.02) | 0.30 | 0.98 |
| EA | CYP24A1 | rs6068816-rs2762939-rs3787555-rs2244719 | G-C-A-A | 0.14 | 0.19 | 0.67 (0.51-0.88) | 0.03 | 0.27 |

*Footnote*: Odds ratio (OR) and 95% confidence interval (CI) are adjusted for covariates including age, proportion of European ancestry, body mass index, family history of breast cancer, and education

**Supplementary Table S3. Risk of estrogen receptor positive breast cancer associated with SNPs in *VDR*, *CYP27B1*, and *CYP24A1*** in African Americana and European American women

| **Gene** | **SNP** | **Genotype** | **African American** | | | |  | **European American** | | | | **Pinteraction** |
| --- | --- | --- | --- | --- | --- | --- | --- | --- | --- | --- | --- | --- |
|  |  |  | **# case/control** | **OR (95% CI)** | **Praw** | **Ppermutation** |  | **# case/control** | **OR (95% CI)** | **Praw** | **Ppermutation** |  |
| VDR | rs10747524 | AA | 100/162 | 1.00 | 0.64 | 1.00 |  | 61/98 | 1.00 | 0.52 | 1.00 | 0.91 |
|  |  | AG | 122/222 | 0.91 (0.65-1.27) |  |  |  | 102/170 | 0.95 (0.63-1.44) |  |  |  |
|  |  | GG | 40/67 | 0.92 (0.57-1.48) |  |  |  | 34/62 | 0.83 (0.48-1.43) |  |  |  |
| VDR | rs11608702 | AA | 155/259 | 1.00 | 0.62 | 1.00 |  | 95/184 | 1.00 | 0.13 | 0.97 | 0.42 |
|  |  | AT | 89/171 | 0.86 (0.62-1.19) |  |  |  | 92/154 | 1.16 (0.8-1.67) |  |  |  |
|  |  | TT | 17/25 | 1.05 (0.54-2.02) |  |  |  | 27/31 | 1.58 (0.88-2.85) |  |  |  |
| VDR | rs12721364 | GG | 251/414 | 1.00 | 0.05 | 0.74 |  | 173/290 | 1.00 | 0.61 | 1.00 | 0.18 |
|  |  | GA/AA | 12/40 | 0.51 (0.26-1) |  |  |  | 41/78 | 0.89 (0.58-1.38) |  |  |  |
| VDR | rs7965281 | AA | 99/151 | 1.00 | 0.07 | 0.94 |  | 52/75 | 1.00 | 0.21 | 1.00 | 0.58 |
|  |  | AG | 134/225 | 0.92 (0.66-1.29) |  |  |  | 113/199 | 0.79 (0.51-1.22) |  |  |  |
|  |  | GG | 30/79 | 0.59 (0.36-0.97) |  |  |  | 48/95 | 0.72 (0.43-1.2) |  |  |  |
| VDR | rs10783215 | AA | 97/179 | 1.00 | 0.96 | 1.00 |  | 56/107 | 1.00 | 0.15 | 1.00 | 0.34 |
|  |  | AG | 128/203 | 1.15 (0.82-1.6) |  |  |  | 105/192 | 1.05 (0.69-1.59) |  |  |  |
|  |  | GG | 38/73 | 0.94 (0.59-1.5) |  |  |  | 53/71 | 1.47 (0.89-2.42) |  |  |  |
| VDR | rs11574143 | GG | 217/386 | 1.00 | 0.36 | 1.00 |  | 164/291 | 1.00 | 0.77 | 1.00 | 0.78 |
|  |  | GA/AA | 46/69 | 1.21 (0.8-1.83) |  |  |  | 50/79 | 1.06 (0.7-1.61) |  |  |  |
| VDR | rs731236 | AA | 127/218 | 1.00 | 0.67 | 1.00 |  | 83/135 | 1.00 | 0.37 | 1.00 | 0.96 |
|  |  | AG | 112/188 | 1.03 (0.74-1.42) |  |  |  | 108/180 | 0.98 (0.67-1.43) |  |  |  |
|  |  | GG | 20/43 | 0.8 (0.45-1.44) |  |  |  | 22/52 | 0.72 (0.4-1.29) |  |  |  |
| VDR | rs7975232 | AA | 130/213 | 1.00 | 0.66 | 1.00 |  | 80/156 | 1.00 | 0.12 | 1.00 | 0.47 |
|  |  | AC | 98/181 | 0.9 (0.64-1.25) |  |  |  | 89/151 | 1.16 (0.79-1.72) |  |  |  |
|  |  | CC | 34/58 | 0.95 (0.59-1.53) |  |  |  | 45/63 | 1.47 (0.91-2.39) |  |  |  |
| VDR | rs1544410 | GG | 118/207 | 1.00 | 0.59 | 1.00 |  | 82/133 | 1.00 | 0.37 | 1.00 | 0.92 |
|  |  | GA | 121/192 | 1.09 (0.79-1.51) |  |  |  | 104/174 | 1 (0.69-1.47) |  |  |  |
|  |  | AA | 22/54 | 0.73 (0.42-1.27) |  |  |  | 28/63 | 0.74 (0.43-1.27) |  |  |  |
| VDR | rs2525044 | GG | 178/301 | 1.00 | 0.9 | 1.00 |  | 66/132 | 1.00 | 0.13 | 1.00 | 0.62 |
|  |  | GA | 72/138 | 0.89 (0.63-1.25) |  |  |  | 106/182 | 1.19 (0.8-1.76) |  |  |  |
|  |  | AA | 13/16 | 1.27 (0.59-2.73) |  |  |  | 41/56 | 1.49 (0.89-2.5) |  |  |  |
| VDR | rs12314197 | AA | 151/281 | 1.00 | 0.23 | 1.00 |  | 213/366 | 1.00 | 0.43 | 1.00 | 0.52 |
|  |  | AG | 93/149 | 1.18 (0.85-1.64) |  |  |  | 1/3 | 0.34 (0.02-4.77) |  |  |  |
|  |  | GG | 18/25 | 1.34 (0.7-2.55) |  |  |  |  |  |  |  |  |
| VDR | rs7963776 | AA | 102/175 | 1.00 | 0.69 | 1.00 |  | 56/114 | 1.00 | 0.15 | 1.00 | 0.47 |
|  |  | AG | 124/209 | 1.02 (0.73-1.42) |  |  |  | 112/190 | 1.2 (0.8-1.81) |  |  |  |
|  |  | GG | 37/71 | 0.88 (0.55-1.41) |  |  |  | 46/66 | 1.45 (0.87-2.42) |  |  |  |
| VDR | rs2239185 | AA | 91/165 | 1.00 | 0.87 | 1.00 |  | 58/114 | 1.00 | 0.25 | 1.00 | 0.78 |
|  |  | AG | 131/215 | 1.1 (0.79-1.54) |  |  |  | 110/189 | 1.13 (0.75-1.7) |  |  |  |
|  |  | GG | 41/73 | 1 (0.63-1.59) |  |  |  | 45/67 | 1.36 (0.81-2.26) |  |  |  |
| VDR | rs7975128 | GG | 131/231 | 1.00 | 0.9 | 1.00 |  | 82/137 | 1.00 | 0.38 | 1.00 | 0.53 |
|  |  | GA | 106/178 | 1.03 (0.74-1.42) |  |  |  | 106/169 | 1.09 (0.75-1.6) |  |  |  |
|  |  | AA | 26/46 | 1.01 (0.6-1.73) |  |  |  | 26/64 | 0.7 (0.4-1.21) |  |  |  |
| VDR | rs11168264 | AA | 155/264 | 1.00 | 1.00 | 1.00 |  | 212/366 | 1.00 | 0.90 | 1.00 | 0.48 |
|  |  | AG | 92/164 | 0.96 (0.69-1.33) |  |  |  | 2/2 | 0.84 (0.06-12.58) |  |  |  |
|  |  | GG | 16/26 | 1.09 (0.56-2.12) |  |  |  |  |  |  |  |  |
| VDR | rs11168266 | AA | 94/169 | 1.00 | 0.65 | 1.00 |  | 60/126 | 1.00 | 0.15 | 1.00 | 0.74 |
|  |  | AG | 126/218 | 1.03 (0.74-1.45) |  |  |  | 109/180 | 1.27 (0.85-1.9) |  |  |  |
|  |  | GG | 42/67 | 1.12 (0.7-1.78) |  |  |  | 44/64 | 1.43 (0.86-2.38) |  |  |  |
| VDR | rs11168268 | AA | 105/196 | 1.00 | 0.47 | 1.00 |  | 62/125 | 1.00 | 0.17 | 1.00 | 0.91 |
|  |  | AG | 125/207 | 1.12 (0.81-1.55) |  |  |  | 108/183 | 1.21 (0.81-1.8) |  |  |  |
|  |  | GG | 33/52 | 1.15 (0.7-1.9) |  |  |  | 43/61 | 1.43 (0.85-2.38) |  |  |  |
| VDR | rs12370156 | AA | 66/120 | 1.00 | 0.99 | 1.00 |  | 48/106 | 1.00 | 0.27 | 1.00 | 0.63 |
|  |  | AG | 137/228 | 1.08 (0.75-1.57) |  |  |  | 117/181 | 1.43 (0.93-2.19) |  |  |  |
|  |  | GG | 59/107 | 0.99 (0.64-1.54) |  |  |  | 49/82 | 1.32 (0.79-2.19) |  |  |  |
| VDR | rs2239182 | GG | 84/151 | 1.00 | 0.46 | 1.00 |  | 53/112 | 1.00 | 0.19 | 1.00 | 0.47 |
|  |  | GA | 126/229 | 0.97 (0.69-1.38) |  |  |  | 113/183 | 1.3 (0.86-1.97) |  |  |  |
|  |  | AA | 51/75 | 1.23 (0.78-1.92) |  |  |  | 48/75 | 1.38 (0.84-2.29) |  |  |  |
| VDR | rs1540339 | GG | 161/263 | 1.00 | 0.64 | 1.00 |  | 84/164 | 1.00 | 0.6 | 1.00 | 0.01 |
|  |  | GA | 83/161 | 0.83 (0.59-1.16) |  |  |  | 96/121 | 1.58 (1.07-2.34) |  |  |  |
|  |  | AA | 17/26 | 1.13 (0.59-2.16) |  |  |  | 18/44 | 0.8 (0.43-1.51) |  |  |  |
| VDR | rs2239179 | AA | 122/196 | 1.00 | 0.31 | 1.00 |  | 66/112 | 1.00 | 0.35 | 1.00 | 0.59 |
|  |  | AG | 113/205 | 0.85 (0.61-1.18) |  |  |  | 112/179 | 1.05 (0.71-1.56) |  |  |  |
|  |  | GG | 27/53 | 0.82 (0.49-1.38) |  |  |  | 36/78 | 0.75 (0.45-1.25) |  |  |  |
| VDR | rs12717991 | GG | 123/204 | 1.00 | 0.56 | 1.00 |  | 94/179 | 1.00 | 0.75 | 1.00 | 0.10 |
|  |  | GA | 113/196 | 0.96 (0.69-1.32) |  |  |  | 101/135 | 1.42 (0.98-2.06) |  |  |  |
|  |  | AA | 27/55 | 0.85 (0.51-1.43) |  |  |  | 19/55 | 0.64 (0.35-1.17) |  |  |  |
| VDR | rs886441 | AA | 91/181 | 1.00 | 0.04 | 0.91 |  | 119/234 | 1.00 | 0.15 | 1.00 | 0.09 |
|  |  | AG | 124/219 | 1.11 (0.79-1.55) |  |  |  | 85/112 | 1.52 (1.05-2.19) |  |  |  |
|  |  | GG | 48/54 | 1.72 (1.08-2.75) |  |  |  | 10/23 | 0.97 (0.44-2.15) |  |  |  |
| VDR | rs2189480 | CC | 112/197 | 1.00 | 0.99 | 1.00 |  | 86/176 | 1.00 | 0.2 | 1.00 | 0.39 |
|  |  | CA | 116/194 | 1.05 (0.76-1.47) |  |  |  | 104/152 | 1.51 (1.04-2.19) |  |  |  |
|  |  | AA | 35/64 | 0.96 (0.59-1.54) |  |  |  | 24/42 | 1.13 (0.63-2.02) |  |  |  |
| VDR | rs3819545 | AA | 148/242 | 1.00 | 0.41 | 1.00 |  | 76/162 | 1.00 | 0.28 | 1.00 | 0.13 |
|  |  | AG | 94/168 | 0.91 (0.66-1.27) |  |  |  | 110/156 | 1.5 (1.03-2.19) |  |  |  |
|  |  | GG | 21/43 | 0.81 (0.46-1.43) |  |  |  | 28/52 | 1.1 (0.64-1.91) |  |  |  |
| VDR | rs3782905 | GG | 153/270 | 1.00 | 0.75 | 1.00 |  | 97/159 | 1.00 | 0.14 | 1.00 | 0.86 |
|  |  | GC | 99/157 | 1.08 (0.78-1.49) |  |  |  | 100/157 | 1.06 (0.73-1.53) |  |  |  |
|  |  | CC | 10/26 | 0.67 (0.31-1.42) |  |  |  | 17/51 | 0.5 (0.27-0.94) |  |  |  |
| VDR | rs2239186 | AA | 240/388 | 1.00 | 0.01 | 0.31 |  | 145/247 | 1.00 | 0.92 | 1.00 | 0.05 |
|  |  | AG/GG | 21/67 | 0.52 (0.31-0.87) |  |  |  | 69/122 | 0.98 (0.68-1.42) |  |  |  |
| VDR | rs10783218 | GG | 178/304 | 1.00 | 0.82 | 1.00 |  | 194/352 | 1.00 | 0.04 | 0.64 | 0.04 |
|  |  | GA/AA | 85/150 | 0.96 (0.69-1.34) |  |  |  | 20/17 | 2.05 (1.02-4.12) |  |  |  |
| VDR | rs10735810 | GG | 159/272 | 1.00 | 0.93 | 1.00 |  | 87/142 | 1.00 | 0.59 | 1.00 | 0.85 |
|  |  | GA | 88/156 | 0.97 (0.7-1.35) |  |  |  | 98/169 | 0.91 (0.63-1.33) |  |  |  |
|  |  | AA | 16/24 | 1.12 (0.57-2.18) |  |  |  | 29/56 | 0.88 (0.52-1.51) |  |  |  |
| VDR | rs11168277 | AA | 186/351 | 1.00 | 0.08 | 1.00 |  | 213/370 |  |  |  |  |
|  |  | AG/GG | 76/103 | 1.37 (0.97-1.94) |  |  |  | 1/0 |  |  |  |  |
| VDR | rs2408876 | AA | 56/124 | 1.00 | 0.15 | 0.99 |  | 77/124 | 1.00 | 0.81 | 1.00 | 0.30 |
|  |  | AG | 139/224 | 1.32 (0.9-1.94) |  |  |  | 98/186 | 0.84 (0.57-1.24) |  |  |  |
|  |  | GG | 68/105 | 1.39 (0.89-2.16) |  |  |  | 39/59 | 1.16 (0.7-1.93) |  |  |  |
| VDR | rs2254210 | GG | 134/202 | 1.00 | 0.23 | 1.00 |  | 92/164 | 1.00 | 0.3 | 1.00 | 0.23 |
|  |  | GA | 101/202 | 0.78 (0.56-1.08) |  |  |  | 94/176 | 0.93 (0.64-1.35) |  |  |  |
|  |  | AA | 27/50 | 0.84 (0.5-1.42) |  |  |  | 28/30 | 1.66 (0.92-3.01) |  |  |  |
| VDR | rs11574044 | AA | 155/250 | 1.00 | 0.35 | 1.00 |  | 161/260 | 1.00 | 0.18 | 1.00 | 0.86 |
|  |  | AC/CC | 108/203 | 0.86 (0.63-1.18) |  |  |  | 53/107 | 0.76 (0.51-1.13) |  |  |  |
| VDR | rs2238136 | GG | 224/377 | 1.00 | 0.49 | 1.00 |  | 111/205 | 1.00 | 0.32 | 1.00 | 0.31 |
|  |  | GA/AA | 39/78 | 0.86 (0.56-1.32) |  |  |  | 102/165 | 1.19 (0.84-1.69) |  |  |  |
| VDR | rs2238135 | GG | 110/208 | 1.00 | 0.40 | 1.00 |  | 119/207 | 1.00 | 0.76 | 1.00 | 0.50 |
|  |  | GC | 124/199 | 1.17 (0.85-1.62) |  |  |  | 84/138 | 1.13 (0.78-1.62) |  |  |  |
|  |  | CC | 29/48 | 1.15 (0.68-1.94) |  |  |  | 10/25 | 0.63 (0.28-1.4) |  |  |  |
| VDR | rs2853564 | AA | 205/352 | 1.00 | 0.85 | 1.00 |  | 89/134 | 1.00 | 0.52 | 1.00 | 0.48 |
|  |  | AG | 55/97 | 0.99 (0.68-1.44) |  |  |  | 89/182 | 0.72 (0.49-1.05) |  |  |  |
|  |  | GG | 3/6 | 0.8 (0.19-3.28) |  |  |  | 35/53 | 0.97 (0.58-1.64) |  |  |  |
| VDR | rs4760648 | AA | 58/95 | 1.00 | 0.44 | 1.00 |  | 69/109 | 1.00 | 0.40 | 1.00 | 0.02 |
|  |  | AG | 138/230 | 0.98 (0.66-1.46) |  |  |  | 93/201 | 0.73 (0.49-1.09) |  |  |  |
|  |  | GG | 67/130 | 0.85 (0.54-1.32) |  |  |  | 52/60 | 1.38 (0.84-2.26) |  |  |  |
| VDR | rs11168287 | AA | 147/253 | 1.00 | 0.92 | 1.00 |  | 50/89 | 1.00 | 0.64 | 1.00 | 1.00 |
|  |  | AG | 94/167 | 1 (0.72-1.39) |  |  |  | 104/179 | 1.01 (0.66-1.57) |  |  |  |
|  |  | GG | 20/33 | 1.05 (0.58-1.91) |  |  |  | 59/100 | 1.12 (0.69-1.82) |  |  |  |
| VDR | rs4328262 | AA | 123/211 | 1.00 | 0.59 | 1.00 |  | 65/128 | 1.00 | 0.30 | 1.00 | 0.69 |
|  |  | AC | 105/194 | 0.97 (0.7-1.35) |  |  |  | 109/173 | 1.19 (0.8-1.77) |  |  |  |
|  |  | CC | 35/50 | 1.23 (0.75-2.01) |  |  |  | 39/64 | 1.29 (0.77-2.15) |  |  |  |
| VDR | rs4334089 | AA | 39/71 | 1.00 | 0.98 | 1.00 |  | 120/217 | 1.00 | 0.89 | 1.00 | 0.95 |
|  |  | AG | 121/208 | 1.03 (0.65-1.63) |  |  |  | 83/133 | 1.12 (0.78-1.61) |  |  |  |
|  |  | GG | 103/176 | 1.02 (0.63-1.63) |  |  |  | 11/20 | 0.81 (0.35-1.89) |  |  |  |
| VDR | rs11168288 | GG | 189/348 | 1.00 | 0.08 | 1.00 |  | 213/370 | 1.00 | 0.99 | 1.00 | 0.97 |
|  |  | GA/AA | 74/105 | 1.37 (0.96-1.94) |  |  |  | 1/0 |  |  |  |  |
| VDR | rs11574026 | GG | 253/436 | 1.00 | 0.74 | 1.00 |  | 168/282 | 1.00 | 0.58 | 1.00 | 0.92 |
|  |  | GA/AA | 10/19 | 0.87 (0.4-1.93) |  |  |  | 46/88 | 0.89 (0.59-1.35) |  |  |  |
| VDR | rs10875695 | CC | 72/124 | 1.00 | 0.71 | 1.00 |  | 117/213 | 1.00 | 0.84 | 1.00 | 0.83 |
|  |  | CA | 123/213 | 0.97 (0.67-1.4) |  |  |  | 83/136 | 1.1 (0.76-1.58) |  |  |  |
|  |  | AA | 66/118 | 0.92 (0.6-1.41) |  |  |  | 13/21 | 0.92 (0.42-2.02) |  |  |  |
| VDR | rs11168292 | GG | 195/327 | 1.00 | 0.6 | 1.00 |  | 104/165 | 1.00 | 0.28 | 1.00 | 0.65 |
|  |  | GC | 57/113 | 0.84 (0.58-1.21) |  |  |  | 88/156 | 0.87 (0.6-1.26) |  |  |  |
|  |  | CC | 10/15 | 1.14 (0.5-2.61) |  |  |  | 22/48 | 0.75 (0.42-1.34) |  |  |  |
| VDR | rs7299460 | AA | 156/255 | 1.00 | 0.61 | 1.00 |  | 18/36 | 1.00 | 0.93 | 1.00 | 0.40 |
|  |  | AG | 90/172 | 0.87 (0.62-1.2) |  |  |  | 97/149 | 1.48 (0.76-2.88) |  |  |  |
|  |  | GG | 17/28 | 1.02 (0.54-1.96) |  |  |  | 98/184 | 1.24 (0.64-2.4) |  |  |  |
| VDR | rs11568820 | GG | 6/26 | 1.00 | 0.04 | 0.48 |  | 123/227 | 1.00 | 0.52 | 1.00 | 0.44 |
|  |  | GA | 73/139 | 2.25 (0.88-5.75) |  |  |  | 79/126 | 1.03 (0.44-2.39) |  |  |  |
|  |  | AA | 184/290 | 2.71 (1.08-6.79) |  |  |  | 12/17 | 0.9 (0.39-2.06) |  |  |  |
| VDR | rs7310552 | AA | 227/383 | 1.00 | 0.56 | 1.00 |  | 84/127 | 1.00 | 0.22 | 1.00 | 0.85 |
|  |  | AG | 34/69 | 0.85 (0.54-1.33) |  |  |  | 99/178 | 0.79 (0.54-1.16) |  |  |  |
|  |  | GG | 2/3 | 1.16 (0.19-7.16) |  |  |  | 31/63 | 0.76 (0.45-1.29) |  |  |  |
| VDR | rs7302038 | GG | 81/135 | 1.00 | 0.84 | 1.00 |  | 209/365 | 1.00 | 0.36 | 0.91 | 0.84 |
|  |  | GA | 127/228 | 0.93 (0.65-1.32) |  |  |  | 3/4 | 1.57 (0.3-8.23) |  |  |  |
|  |  | AA | 55/92 | 0.97 (0.62-1.5) |  |  |  | Feb-00 | 1161481.53 (0-.) |  |  |  |
| VDR | rs4442605 | CC | 118/224 | 1.00 | 0.54 | 1.00 |  | 126/228 | 1.00 | 0.58 | 1.00 | 0.94 |
|  |  | CA | 120/188 | 1.21 (0.88-1.67) |  |  |  | 79/126 | 1.13 (0.78-1.63) |  |  |  |
|  |  | AA | 24/43 | 1 (0.58-1.74) |  |  |  | 9/15 | 1.06 (0.44-2.57) |  |  |  |
| CYP27B1 | rs4646536 | AA | 140/252 | 1.00 | 0.23 | 1.00 |  | 109/190 | 1.00 | 0.98 | 1.00 | 0.35 |
|  |  | AG | 99/182 | 0.97 (0.7-1.33) |  |  |  | 85/147 | 0.99 (0.68-1.42) |  |  |  |
|  |  | GG | 24/21 | 1.94 (1.03-3.64) |  |  |  | 20/33 | 1.03 (0.56-1.91) |  |  |  |
| CYP24A1 | rs11907350 | GG | 170/314 | 1.00 | 0.34 | 1.00 |  | 203/336 | 1.00 | 0.15 | 0.97 | 0.09 |
|  |  | GA/AA | 92/141 | 1.17 (0.85-1.63) |  |  |  | 11/33 | 0.58 (0.28-1.21) |  |  |  |
| CYP24A1 | rs927650 | GG | 145/258 | 1.00 | 0.92 | 1.00 |  | 60/116 | 1.00 | 0.37 | 1.00 | 0.84 |
|  |  | GA | 103/170 | 1.06 (0.77-1.47) |  |  |  | 111/186 | 1.18 (0.79-1.77) |  |  |  |
|  |  | AA | 15/27 | 0.93 (0.48-1.83) |  |  |  | 41/68 | 1.25 (0.75-2.09) |  |  |  |
| CYP24A1 | rs1570669 | GG | 47/81 | 1.00 | 0.58 | 1.00 |  | 93/158 | 1.00 | 0.22 | 1.00 | 0.15 |
|  |  | GA | 122/219 | 1 (0.65-1.54) |  |  |  | 104/162 | 1.09 (0.76-1.57) |  |  |  |
|  |  | AA | 94/154 | 1.11 (0.71-1.75) |  |  |  | 17/50 | 0.53 (0.28-1.01) |  |  |  |
| CYP24A1 | rs912505 | AA | 78/138 | 1.00 | 0.40 | 1.00 |  | 126/208 | 1.00 | 0.18 | 1.00 | 0.26 |
|  |  | AG | 132/240 | 0.99 (0.69-1.41) |  |  |  | 79/136 | 0.94 (0.65-1.35) |  |  |  |
|  |  | GG | 52/76 | 1.25 (0.8-1.98) |  |  |  | 9/26 | 0.48 (0.21-1.11) |  |  |  |
| CYP24A1 | rs2209314 | AA | 213/393 | 1.00 | 0.05 | 1.00 |  | 109/208 | 1.00 | 0.31 | 1.00 | 0.42 |
|  |  | AG/GG | 49/61 | 1.52 (1-2.32) |  |  |  | 105/162 | 1.2 (0.85-1.7) |  |  |  |
| CYP24A1 | rs6068816 | GG | 239/417 | 1.00 | 0.70 | 1.00 |  | 159/289 | 1.00 | 0.45 | 1.00 | 0.97 |
|  |  | GA/AA | 24/38 | 1.11 (0.65-1.92) |  |  |  | 55/81 | 1.17 (0.78-1.76) |  |  |  |
| CYP24A1 | rs2762939 | GG | 46/94 | 1.00 | 0.44 | 1.00 |  | 106/202 | 1.00 | 0.47 | 1.00 | 0.61 |
|  |  | GC | 146/206 | 1.43 (0.94-2.17) |  |  |  | 92/143 | 1.22 (0.85-1.76) |  |  |  |
|  |  | CC | 70/154 | 0.92 (0.58-1.46) |  |  |  | 15/24 | 1.03 (0.5-2.11) |  |  |  |
| CYP24A1 | rs3787555 | CC | 183/326 | 1.00 | 0.55 | 1.00 |  | 114/175 | 1.00 | 0.08 | 1.00 | 0.23 |
|  |  | CA | 75/122 | 1.08 (0.76-1.52) |  |  |  | 83/155 | 0.78 (0.54-1.13) |  |  |  |
|  |  | AA | 3/7 | 1.36 (0.42-4.43) |  |  |  | 17/38 | 0.63 (0.33-1.18) |  |  |  |
| CYP24A1 | rs2244719 | AA | 135/267 | 1.00 | 0.21 | 1.00 |  | 73/134 | 1.00 | 0.17 | 1.00 | 0.32 |
|  |  | AG | 107/155 | 1.38 (0.99-1.91) |  |  |  | 96/174 | 1.06 (0.71-1.57) |  |  |  |
|  |  | GG | 17/32 | 1.04 (0.55-1.95) |  |  |  | 42/59 | 1.47 (0.89-2.44) |  |  |  |
| CYP24A1 | rs2762941 | AA | 68/120 | 1.00 | 0.45 | 1.00 |  | 66/132 | 1.00 | 0.52 | 1.00 | 0.59 |
|  |  | AG | 139/212 | 1.17 (0.8-1.69) |  |  |  | 107/168 | 1.26 (0.85-1.87) |  |  |  |
|  |  | GG | 56/122 | 0.84 (0.54-1.3) |  |  |  | 41/68 | 1.12 (0.67-1.85) |  |  |  |
| CYP24A1 | rs2181874 | GG | 96/164 | 1.00 | 0.44 | 1.00 |  | 109/204 | 1.00 | 0.43 | 1.00 | 0.68 |
|  |  | GA | 134/212 | 1.11 (0.79-1.55) |  |  |  | 92/142 | 1.29 (0.9-1.86) |  |  |  |
|  |  | AA | 33/77 | 0.75 (0.46-1.21) |  |  |  | 12/23 | 0.92 (0.42-2) |  |  |  |
| CYP24A1 | rs4809959 | GG | 60/133 | 1.00 | 0.32 | 1.00 |  | 65/131 | 1.00 | 0.11 | 1.00 | 0.48 |
|  |  | GA | 146/223 | 1.48 (1.02-2.14) |  |  |  | 106/172 | 1.24 (0.83-1.84) |  |  |  |
|  |  | AA | 57/98 | 1.22 (0.78-1.92) |  |  |  | 43/66 | 1.5 (0.9-2.49) |  |  |  |
| CYP24A1 | rs2245153 | AA | 108/166 | 1.00 | 0.12 | 1.00 |  | 121/194 | 1.00 | 0.16 | 1.00 | 0.39 |
|  |  | AG | 126/208 | 0.93 (0.67-1.3) |  |  |  | 72/139 | 0.75 (0.51-1.09) |  |  |  |
|  |  | GG | 17/49 | 0.55 (0.3-1.01) |  |  |  | 13/25 | 0.77 (0.37-1.64) |  |  |  |
| CYP24A1 | rs2585428 | GG | 67/109 | 1.00 | 0.70 | 1.00 |  | 54/90 | 1.00 | 0.64 | 1.00 | 0.97 |
|  |  | GA | 140/246 | 0.99 (0.68-1.44) |  |  |  | 101/175 | 0.95 (0.61-1.46) |  |  |  |
|  |  | AA | 54/100 | 0.91 (0.58-1.44) |  |  |  | 59/105 | 0.89 (0.55-1.44) |  |  |  |
| CYP24A1 | rs6022999 | GG | 33/55 | 1.00 | 0.52 | 1.00 |  | 112/200 | 1.00 | 0.84 | 1.00 | 0.78 |
|  |  | GA | 127/207 | 1.05 (0.64-1.71) |  |  |  | 86/132 | 1.17 (0.81-1.69) |  |  |  |
|  |  | AA | 102/192 | 0.91 (0.55-1.51) |  |  |  | 16/38 | 0.73 (0.38-1.42) |  |  |  |

*Footnote*: Odds ratio (OR) and 95% confidence interval (CI) are adjusted for covariates including age, proportion of European ancestry, body mass index, family history of breast cancer, and education. P­trend was for genetic dose-response by coding genotypes as 0, 1 and 2 based on the number of variant allele. Pinteraction was for the differences in odds ratios between pre- and postmenopausal women.

**Supplementary Table S4. Risk of estrogen receptor negative breast cancer associated with SNPs in *VDR*, *CYP27B1*, and *CYP24A1* in African Americana and European American women**

| **Gene** | **SNP** | **Genotype** | **African American** | | | |  | **European American** | | | | **Pinteraction** |
| --- | --- | --- | --- | --- | --- | --- | --- | --- | --- | --- | --- | --- |
|  |  |  | **# case/control** | **OR (95% CI)** | **Praw** | **Ppermutation** |  | **# case/control** | **OR (95% CI)** | **Praw** | **Ppermutation** |  |
| VDR | rs10747524 | AA | 43/162 | 1.00 | 0.53 | 1.00 |  | 14/98 | 1.00 | 0.16 | 1.00 | 0.91 |
|  |  | AG | 52/222 | 0.9 (0.57-1.43) |  |  |  | 25/170 | 1.1 (0.54-2.25) |  |  |  |
|  |  | GG | 24/67 | 1.31 (0.72-2.38) |  |  |  | 15/62 | 1.82 (0.81-4.12) |  |  |  |
| VDR | rs11608702 | AA | 79/259 | 1.00 | 0.32 | 1.00 |  | 25/184 | 1.00 | 0.07 | 0.85 | 0.27 |
|  |  | AT | 33/171 | 0.63 (0.4-1) |  |  |  | 23/154 | 1.09 (0.59-2.01) |  |  |  |
|  |  | TT | 9/25 | 1.21 (0.53-2.76) |  |  |  | 11/31 | 2.51 (1.11-5.69) |  |  |  |
| VDR | rs12721364 | GG | 117/414 | 1.00 | 0.05 | 0.64 |  | 44/290 | 1.00 | 0.53 | 1.00 | 0.04 |
|  |  | GA/AA | 3/40 | 0.29 (0.09-0.98) |  |  |  | 15/78 | 1.23 (0.64-2.35) |  |  |  |
| VDR | rs7965281 | AA | 47/151 | 1.00 | 0.97 | 1.00 |  | 17/75 | 1.00 | 0.27 | 1.00 | 0.51 |
|  |  | AG | 49/225 | 0.72 (0.46-1.14) |  |  |  | 28/199 | 0.61 (0.31-1.19) |  |  |  |
|  |  | GG | 25/79 | 1.14 (0.64-2.02) |  |  |  | 14/95 | 0.64 (0.29-1.41) |  |  |  |
| VDR | rs10783215 | AA | 46/179 | 1.00 | 0.75 | 1.00 |  | 15/107 | 1.00 | 0.28 | 1.00 | 0.85 |
|  |  | AG | 53/203 | 0.95 (0.6-1.5) |  |  |  | 29/192 | 1.04 (0.53-2.05) |  |  |  |
|  |  | GG | 22/73 | 1.15 (0.64-2.07) |  |  |  | 15/71 | 1.58 (0.71-3.51) |  |  |  |
| VDR | rs11574143 | GG | 104/386 | 1.00 | 0.63 | 1.00 |  | 46/291 | 1.00 | 0.98 | 1.00 | 0.68 |
|  |  | GA/AA | 17/69 | 0.87 (0.48-1.55) |  |  |  | 13/79 | 1.01 (0.51-1.98) |  |  |  |
| VDR | rs731236 | AA | 62/218 | 1.00 | 0.58 | 1.00 |  | 21/135 | 1.00 | 0.8 | 1.00 | 0.76 |
|  |  | AG | 47/188 | 0.9 (0.58-1.39) |  |  |  | 32/180 | 1.13 (0.62-2.06) |  |  |  |
|  |  | GG | 9/43 | 0.85 (0.39-1.89) |  |  |  | 6/52 | 0.77 (0.29-2.03) |  |  |  |
| VDR | rs7975232 | AA | 60/213 | 1.00 | 0.74 | 1.00 |  | 18/156 | 1.00 | 0.08 | 0.95 | 0.26 |
|  |  | AC | 46/181 | 0.95 (0.61-1.48) |  |  |  | 28/151 | 1.64 (0.85-3.14) |  |  |  |
|  |  | CC | 15/58 | 0.91 (0.47-1.73) |  |  |  | 13/63 | 1.94 (0.88-4.29) |  |  |  |
| VDR | rs1544410 | GG | 60/207 | 1.00 | 0.97 | 1.00 |  | 22/133 | 1.00 | 0.48 | 1.00 | 0.3 |
|  |  | GA | 46/192 | 0.87 (0.56-1.36) |  |  |  | 31/174 | 1.09 (0.6-2) |  |  |  |
|  |  | AA | 15/54 | 1.11 (0.58-2.15) |  |  |  | 6/63 | 0.61 (0.23-1.59) |  |  |  |
| VDR | rs2525044 | GG | 77/301 | 1.00 | 0.52 | 1.00 |  | 17/132 | 1.00 | 0.24 | 1.00 | 0.95 |
|  |  | GA | 39/138 | 1.14 (0.73-1.79) |  |  |  | 30/182 | 1.32 (0.69-2.53) |  |  |  |
|  |  | AA | 5/16 | 1.22 (0.42-3.52) |  |  |  | 11/56 | 1.62 (0.7-3.73) |  |  |  |
| VDR | rs12314197 | AA | 65/281 | 1.00 | 0.63 | 1.00 |  | 58/366 | 1.00 | 0.76 | 1.00 | 0.8 |
|  |  | AG | 52/149 | 1.41 (0.92-2.16) |  |  |  | 1/3 | 1.48 (0.12-17.89) |  |  |  |
|  |  | GG | 4/25 | 0.58 (0.19-1.75) |  |  |  |  |  |  |  |  |
| VDR | rs7963776 | AA | 41/175 | 1.00 | 0.49 | 1.00 |  | 13/114 | 1.00 | 0.15 | 1.00 | 0.72 |
|  |  | AG | 62/209 | 1.27 (0.81-2) |  |  |  | 33/190 | 1.52 (0.75-3.06) |  |  |  |
|  |  | GG | 18/71 | 1.15 (0.61-2.15) |  |  |  | 13/66 | 1.84 (0.79-4.27) |  |  |  |
| VDR | rs2239185 | AA | 44/165 | 1.00 | 0.35 | 1.00 |  | 13/114 | 1.00 | 0.12 | 1.00 | 0.47 |
|  |  | AG | 50/215 | 0.86 (0.54-1.36) |  |  |  | 31/189 | 1.46 (0.72-2.95) |  |  |  |
|  |  | GG | 27/73 | 1.44 (0.82-2.55) |  |  |  | 14/67 | 1.93 (0.84-4.45) |  |  |  |
| VDR | rs7975128 | GG | 65/231 | 1.00 | 0.97 | 1.00 |  | 22/137 | 1.00 | 0.54 | 1.00 | 0.37 |
|  |  | GA | 44/178 | 0.92 (0.59-1.43) |  |  |  | 31/169 | 1.16 (0.64-2.12) |  |  |  |
|  |  | AA | 12/46 | 1.08 (0.53-2.2) |  |  |  | 6/64 | 0.62 (0.24-1.62) |  |  |  |
| VDR | rs11168264 | AA | 66/264 | 1.00 | 0.79 | 1.00 |  | 57/366 | 1.00 | 0.16 | 0.79 | 0.11 |
|  |  | AG | 50/164 | 1.1 (0.72-1.7) |  |  |  | 2/2 | 4.8 (0.53-43.67) |  |  |  |
|  |  | GG | 4/26 | 0.59 (0.19-1.76) |  |  |  |  |  |  |  |  |
| VDR | rs11168266 | AA | 41/169 | 1.00 | 0.24 | 1.00 |  | 16/126 | 1.00 | 0.30 | 1.00 | 0.59 |
|  |  | AG | 54/218 | 0.95 (0.6-1.51) |  |  |  | 31/180 | 1.34 (0.7-2.59) |  |  |  |
|  |  | GG | 26/67 | 1.53 (0.86-2.73) |  |  |  | 12/64 | 1.51 (0.66-3.43) |  |  |  |
| VDR | rs11168268 | AA | 49/196 | 1.00 | 0.55 | 1.00 |  | 17/125 | 1.00 | 0.34 | 1.00 | 0.91 |
|  |  | AG | 54/207 | 1.01 (0.65-1.57) |  |  |  | 30/183 | 1.22 (0.64-2.33) |  |  |  |
|  |  | GG | 18/52 | 1.28 (0.68-2.42) |  |  |  | 12/61 | 1.49 (0.66-3.36) |  |  |  |
| VDR | rs12370156 | AA | 27/120 | 1.00 | 0.63 | 1.00 |  | 11/106 | 1.00 | 0.23 | 1.00 | 0.68 |
|  |  | AG | 64/228 | 1.2 (0.72-2) |  |  |  | 34/181 | 1.78 (0.86-3.7) |  |  |  |
|  |  | GG | 29/107 | 1.16 (0.64-2.1) |  |  |  | 14/82 | 1.68 (0.71-3.93) |  |  |  |
| VDR | rs2239182 | GG | 31/151 | 1.00 | 0.51 | 1.00 |  | 11/112 | 1.00 | 0.09 | 0.96 | 0.42 |
|  |  | GA | 72/229 | 1.52 (0.94-2.44) |  |  |  | 32/183 | 1.86 (0.89-3.88) |  |  |  |
|  |  | AA | 17/75 | 1.08 (0.56-2.11) |  |  |  | 16/75 | 2.04 (0.89-4.71) |  |  |  |
| VDR | rs1540339 | GG | 74/263 | 1.00 | 0.18 | 1.00 |  | 20/164 | 1.00 | 0.18 | 0.99 | 0.06 |
|  |  | GA | 42/161 | 0.95 (0.61-1.46) |  |  |  | 25/121 | 1.62 (0.85-3.09) |  |  |  |
|  |  | AA | 2/26 | 0.26 (0.06-1.13) |  |  |  | 9/44 | 1.57 (0.66-3.73) |  |  |  |
| VDR | rs2239179 | AA | 53/196 | 1.00 | 0.61 | 1.00 |  | 20/112 | 1.00 | 0.09 | 0.98 | 0.51 |
|  |  | AG | 60/205 | 1.12 (0.73-1.73) |  |  |  | 35/179 | 1.11 (0.6-2.03) |  |  |  |
|  |  | GG | 8/53 | 0.63 (0.28-1.42) |  |  |  | 4/78 | 0.3 (0.1-0.91) |  |  |  |
| VDR | rs12717991 | GG | 55/204 | 1.00 | 0.69 | 1.00 |  | 22/179 | 1.00 | 0.43 | 1.00 | 0.48 |
|  |  | GA | 55/196 | 1.04 (0.67-1.59) |  |  |  | 26/135 | 1.49 (0.8-2.77) |  |  |  |
|  |  | AA | 11/55 | 0.78 (0.38-1.62) |  |  |  | 9/55 | 1.21 (0.52-2.82) |  |  |  |
| VDR | rs886441 | AA | 38/181 | 1.00 | 0.20 | 1.00 |  | 34/234 | 1.00 | 0.61 | 1.00 | 0.6 |
|  |  | AG | 65/219 | 1.33 (0.84-2.09) |  |  |  | 23/112 | 1.43 (0.79-2.56) |  |  |  |
|  |  | GG | 18/54 | 1.43 (0.75-2.73) |  |  |  | 2/23 | 0.69 (0.15-3.12) |  |  |  |
| VDR | rs2189480 | CC | 46/197 | 1.00 | 0.84 | 1.00 |  | 23/176 | 1.00 | 0.18 | 0.99 | 0.17 |
|  |  | CA | 64/194 | 1.41 (0.91-2.18) |  |  |  | 26/152 | 1.36 (0.74-2.51) |  |  |  |
|  |  | AA | 11/64 | 0.66 (0.32-1.37) |  |  |  | 10/42 | 1.68 (0.73-3.88) |  |  |  |
| VDR | rs3819545 | AA | 71/242 | 1.00 | 0.04 | 0.89 |  | 22/162 | 1.00 | 0.38 | 1.00 | 0.04 |
|  |  | AG | 45/168 | 0.91 (0.59-1.4) |  |  |  | 26/156 | 1.22 (0.66-2.26) |  |  |  |
|  |  | GG | 3/43 | 0.23 (0.07-0.77) |  |  |  | 11/52 | 1.4 (0.62-3.15) |  |  |  |
| VDR | rs3782905 | GG | 71/270 | 1.00 | 0.95 | 1.00 |  | 32/159 | 1.00 | 0.03 | 0.56 | 0.22 |
|  |  | GC | 44/157 | 1.1 (0.71-1.69) |  |  |  | 25/157 | 0.79 (0.44-1.41) |  |  |  |
|  |  | CC | 5/26 | 0.83 (0.3-2.27) |  |  |  | 2/54 | 0.19 (0.04-0.84) |  |  |  |
| VDR | rs2239186 | AA | 109/388 | 1.00 | 0.15 | 0.97 |  | 40/247 | 1.00 | 0.82 | 1.00 | 0.39 |
|  |  | AG/GG | Nov-67 | 0.61 (0.31-1.2) |  |  |  | 19/122 | 0.93 (0.51-1.69) |  |  |  |
| VDR | rs10783218 | GG | 74/304 | 1.00 | 0.32 | 1.00 |  | 57/352 | 1.00 | 0.55 | 1.00 | 0.46 |
|  |  | GA/AA | 47/150 | 1.24 (0.81-1.9) |  |  |  | 2/17 | 0.63 (0.13-2.92) |  |  |  |
| VDR | rs10735810 | GG | 73/272 | 1.00 | 0.84 | 1.00 |  | 26/142 | 1.00 | 0.24 | 1.00 | 0.59 |
|  |  | GA | 39/156 | 0.94 (0.6-1.46) |  |  |  | 26/169 | 0.78 (0.42-1.42) |  |  |  |
|  |  | AA | 8/24 | 1.31 (0.56-3.09) |  |  |  | 7/56 | 0.61 (0.24-1.52) |  |  |  |
| VDR | rs11168277 | AA | 86/351 | 1.00 | 0.46 | 1.00 |  | 59/370 |  |  |  |  |
|  |  | AG/GG | 33/103 | 1.19 (0.75-1.9) |  |  |  |  |  |  |  |  |
| VDR | rs2408876 | AA | 33/124 | 1.00 | 0.75 | 1.00 |  | 17/124 | 1.00 | 0.27 | 1.00 | 0.37 |
|  |  | AG | 63/224 | 1.07 (0.66-1.74) |  |  |  | 29/186 | 1.07 (0.56-2.06) |  |  |  |
|  |  | GG | 25/105 | 0.9 (0.49-1.63) |  |  |  | 13/59 | 1.64 (0.74-3.62) |  |  |  |
| VDR | rs2254210 | GG | 56/202 | 1.00 | 0.79 | 1.00 |  | 32/164 | 1.00 | 0.33 | 1.00 | 0.68 |
|  |  | GA | 50/202 | 0.87 (0.56-1.35) |  |  |  | 22/176 | 0.66 (0.36-1.19) |  |  |  |
|  |  | AA | 15/50 | 1.01 (0.52-1.95) |  |  |  | 5/30 | 0.88 (0.31-2.46) |  |  |  |
| VDR | rs11574044 | AA | 69/250 | 1.00 | 0.54 | 1.00 |  | 39/260 | 1.00 | 0.46 | 1.00 | 0.34 |
|  |  | AC/CC | 51/203 | 0.88 (0.58-1.33) |  |  |  | 20/107 | 1.25 (0.69-2.26) |  |  |  |
| VDR | rs2238136 | GG | 107/377 | 1.00 | 0.30 | 1.00 |  | 31/205 | 1.00 | 0.68 | 1.00 | 0.26 |
|  |  | GA/AA | 14/78 | 0.72 (0.39-1.34) |  |  |  | 28/165 | 1.13 (0.64-1.96) |  |  |  |
| VDR | rs2238135 | GG | 66/208 | 1.00 | 0.13 | 1.00 |  | 29/207 | 1.00 | 0.26 | 1.00 | 0.17 |
|  |  | GC | 44/199 | 0.71 (0.46-1.1) |  |  |  | 24/138 | 1.28 (0.71-2.31) |  |  |  |
|  |  | CC | 11/48 | 0.7 (0.34-1.44) |  |  |  | 6/25 | 1.62 (0.6-4.38) |  |  |  |
| VDR | rs2853564 | AA | 95/352 | 1.00 | 0.9 | 1.00 |  | 28/134 | 1.00 | 0.08 | 0.96 | 0.31 |
|  |  | AG | 23/97 | 0.96 (0.57-1.61) |  |  |  | 26/182 | 0.69 (0.38-1.24) |  |  |  |
|  |  | GG | 6-Feb | 1.69 (0.31-9.17) |  |  |  | 5/53 | 0.45 (0.16-1.23) |  |  |  |
| VDR | rs4760648 | AA | 23/95 | 1.00 | 0.63 | 1.00 |  | 14/109 | 1.00 | 0.40 | 1.00 | 0.92 |
|  |  | AG | 60/230 | 1.11 (0.64-1.92) |  |  |  | 34/201 | 1.27 (0.65-2.49) |  |  |  |
|  |  | GG | 37/130 | 1.16 (0.64-2.11) |  |  |  | 11/60 | 1.43 (0.6-3.39) |  |  |  |
| VDR | rs11168287 | AA | 63/253 | 1.00 | 0.19 | 1.00 |  | 15/89 | 1.00 | 0.68 | 1.00 | 0.69 |
|  |  | AG | 46/167 | 1.17 (0.75-1.82) |  |  |  | 26/179 | 0.92 (0.46-1.86) |  |  |  |
|  |  | GG | 12/33 | 1.63 (0.78-3.4) |  |  |  | 18/100 | 1.16 (0.54-2.48) |  |  |  |
| VDR | rs4328262 | AA | 62/211 | 1.00 | 0.46 | 1.00 |  | 19/128 | 1.00 | 0.50 | 1.00 | 0.65 |
|  |  | AC | 47/194 | 0.82 (0.53-1.27) |  |  |  | 28/173 | 1.15 (0.61-2.17) |  |  |  |
|  |  | CC | 12/50 | 0.87 (0.43-1.76) |  |  |  | 12/64 | 1.31 (0.59-2.91) |  |  |  |
| VDR | rs4334089 | AA | 15/71 | 1.00 | 0.95 | 1.00 |  | 33/217 | 1.00 | 0.79 | 1.00 | 0.97 |
|  |  | AG | 58/208 | 1.21 (0.64-2.29) |  |  |  | 23/133 | 1.11 (0.62-1.99) |  |  |  |
|  |  | GG | 48/176 | 1.07 (0.55-2.07) |  |  |  | 3/20 | 1.02 (0.27-3.78) |  |  |  |
| VDR | rs11168288 | GG | 91/348 | 1.00 | 0.65 | 1.00 |  | 59/370 |  | . |  |  |
|  |  | GA/AA | 30/105 | 1.12 (0.69-1.8) |  |  |  |  |  |  |  |  |
| VDR | rs11574026 | GG | 116/436 | 1.00 | 0.55 | 1.00 |  | 47/282 | 1.00 | 0.64 | 1.00 | 0.40 |
|  |  | GA/AA | 9/19 | 1.38 (0.49-3.91) |  |  |  | 12/88 | 0.85 (0.43-1.68) |  |  |  |
| VDR | rs10875695 | CC | 27/124 | 1.00 | 0.91 | 1.00 |  | 33/213 | 1.00 | 0.91 | 1.00 | 0.82 |
|  |  | CA | 66/213 | 1.35 (0.81-2.25) |  |  |  | 23/136 | 1.09 (0.61-1.95) |  |  |  |
|  |  | AA | 28/118 | 0.97 (0.53-1.77) |  |  |  | 5/21 | 0.92 (0.25-3.36) |  |  |  |
| VDR | rs11168292 | GG | 91/327 | 1.00 | 0.42 | 1.00 |  | 27/165 | 1.00 | 0.54 | 1.00 | 0.78 |
|  |  | GC | 29/113 | 0.95 (0.59-1.53) |  |  |  | 27/156 | 1.08 (0.6-1.95) |  |  |  |
|  |  | CC | 1/15 | 0.3 (0.04-2.35) |  |  |  | 5/48 | 0.61 (0.22-1.69) |  |  |  |
| VDR | rs7299460 | AA | 80/255 | 1.00 | 0.14 | 0.92 |  | 5/36 | 1.00 | 0.91 | 1.00 | 0.63 |
|  |  | AG | 36/172 | 0.71 (0.45-1.11) |  |  |  | 24/149 | 1.17 (0.4-3.39) |  |  |  |
|  |  | GG | 5/28 | 0.72 (0.26-1.97) |  |  |  | 29/184 | 1.13 (0.4-3.22) |  |  |  |
| VDR | rs11568820 | GG | 6/26 | 1.00 | 0.26 | 0.97 |  | 38/227 | 1.00 | 0.26 | 1.00 | 0.85 |
|  |  | GA | 26/139 | 0.70 (0.26-1.90) |  |  |  | 21/126 | 0.91 (0.51-1.65) |  |  |  |
|  |  | AA | 89/290 | 1.06 (0.41-2.73) |  |  |  | 0/17 |  |  |  |  |
| VDR | rs7310552 | AA | 105/383 | 1.00 | 0.80 | 1.00 |  | 21/127 | 1.00 | 0.94 | 1.00 | 0.32 |
|  |  | AG | 14/69 | 0.86 (0.46-1.63) |  |  |  | 28/178 | 0.95 (0.51-1.76) |  |  |  |
|  |  | GG | 2/3 | 4.54 (0.69-30.13) |  |  |  | 10/63 | 0.99 (0.43-2.26) |  |  |  |
| VDR | rs7302038 | GG | 33/135 | 1.00 | 0.96 | 1.00 |  | 57/365 | 1.00 | 0.32 | 1.00 | 0.23 |
|  |  | GA | 61/228 | 0.96 (0.59-1.57) |  |  |  | 2/4 | 2.9 (0.36-23.3) |  |  |  |
|  |  | AA | 27/92 | 0.99 (0.54-1.8) |  |  |  |  |  |  |  |  |
| VDR | rs4442605 | CC | 62/224 | 1.00 | 0.97 | 1.00 |  | 40/228 | 1.00 | 0.17 | 0.98 | 1.00 |
|  |  | CA | 45/188 | 0.84 (0.54-1.31) |  |  |  | 19/126 | 0.81 (0.44-1.47) |  |  |  |
|  |  | AA | 14/43 | 1.23 (0.62-2.43) |  |  |  | 0/15 |  |  |  |  |
| CYP27B1 | rs4646536 | AA | 69/252 | 1.00 | 0.76 | 1.00 |  | 29/190 | 1.00 | 0.58 | 1.00 | 0.86 |
|  |  | AG | 46/182 | 0.89 (0.58-1.36) |  |  |  | 23/147 | 1.08 (0.59-1.97) |  |  |  |
|  |  | GG | 6/21 | 1.08 (0.41-2.84) |  |  |  | 7/33 | 1.31 (0.52-3.29) |  |  |  |
| CYP24A1 | rs11907350 | GG | 87/314 | 1.00 | 0.32 | 1.00 |  | 57/336 | 1.00 | 0.18 | 0.99 | 0.33 |
|  |  | GA/AA | 34/141 | 0.79 (0.5-1.25) |  |  |  | 2/33 | 0.37 (0.09-1.58) |  |  |  |
| CYP24A1 | rs927650 | GG | 66/258 | 1.00 | 0.62 | 1.00 |  | 10/116 | 1.00 | 0.003 | 0.15 | 0.20 |
|  |  | GA | 47/170 | 1.09 (0.71-1.68) |  |  |  | 29/186 | 1.76 (0.81-3.78) |  |  |  |
|  |  | AA | 8/27 | 1.18 (0.5-2.79) |  |  |  | 20/68 | 3.46 (1.5-7.96) |  |  |  |
| CYP24A1 | rs1570669 | GG | 15/81 | 1.00 | 0.69 | 1.00 |  | 32/158 | 1.00 | 0.03 | 0.92 | 0.05 |
|  |  | GA | 69/219 | 1.77 (0.94-3.31) |  |  |  | 24/162 | 0.71 (0.4-1.27) |  |  |  |
|  |  | AA | 37/154 | 1.36 (0.69-2.67) |  |  |  | 3/50 | 0.28 (0.08-0.97) |  |  |  |
| CYP24A1 | rs912505 | AA | 36/138 | 1.00 | 0.97 | 1.00 |  | 43/208 | 1.00 | 0.01 | 0.49 | 0.09 |
|  |  | AG | 66/240 | 1.11 (0.69-1.78) |  |  |  | 15/136 | 0.52 (0.28-0.98) |  |  |  |
|  |  | GG | 19/76 | 0.98 (0.51-1.85) |  |  |  | 6/26 | 0.18 (0.02-1.38) |  |  |  |
| CYP24A1 | rs2209314 | AA | 102/393 | 1.00 | 0.33 | 1.00 |  | 46/208 | 1.00 | 0.004 | 0.07 | 0.003 |
|  |  | AG/GG | 19/61 | 1.34 (0.74-2.4) |  |  |  | 13/162 | 0.38 (0.2-0.73) |  |  |  |
| CYP24A1 | rs6068816 | GG | 109/417 | 1.00 | 0.56 | 1.00 |  | 43/289 | 1.00 | 0.44 | 1.00 | 0.94 |
|  |  | GA/AA | 12/38 | 1.23 (0.61-2.48) |  |  |  | 16/81 | 1.28 (0.68-2.42) |  |  |  |
| CYP24A1 | rs2762939 | GG | 25/94 | 1.00 | 0.48 | 1.00 |  | 37/202 | 1.00 | 0.13 | 0.82 | 1.00 |
|  |  | GC | 55/206 | 0.85 (0.49-1.48) |  |  |  | 22/143 | 0.86 (0.48-1.53) |  |  |  |
|  |  | CC | 40/154 | 0.8 (0.45-1.44) |  |  |  | 0/24 |  |  |  |  |
| CYP24A1 | rs3787555 | CC | 79/326 | 1.00 | 0.02 | 0.98 |  | 25/175 | 1.00 | 0.91 | 1.00 | 0.19 |
|  |  | CA | 37/122 | 1.42 (0.9-2.24) |  |  |  | 29/155 | 1.25 (0.69-2.24) |  |  |  |
|  |  | AA | 5/7 | 3.79 (1.11-12.91) |  |  |  | 5/38 | 0.83 (0.3-2.35) |  |  |  |
| CYP24A1 | rs2244719 | AA | 76/267 | 1.00 | 0.38 | 1.00 |  | 15/134 | 1.00 | 0.3 | 1.00 | 0.18 |
|  |  | AG | 39/155 | 0.9 (0.57-1.41) |  |  |  | 35/174 | 1.73 (0.9-3.33) |  |  |  |
|  |  | GG | 5/32 | 0.64 (0.24-1.73) |  |  |  | 9/59 | 1.37 (0.56-3.34) |  |  |  |
| CYP24A1 | rs2762941 | AA | 16/120 | 1.00 | 0.004 | 0.09 |  | 24/132 | 1.00 | 0.54 | 1.00 | 0.05 |
|  |  | AG | 61/212 | 1.97 (1.07-3.61) |  |  |  | 26/168 | 0.89 (0.49-1.65) |  |  |  |
|  |  | GG | 44/122 | 2.62 (1.38-4.98) |  |  |  | Sep-68 | 0.78 (0.34-1.78) |  |  |  |
| CYP24A1 | rs2181874 | GG | 33/164 | 1.00 | 0.22 | 1.00 |  | 40/204 | 1.00 | 0.26 | 0.99 | 0.08 |
|  |  | GA | 65/212 | 1.41 (0.88-2.28) |  |  |  | 15/142 | 0.56 (0.29-1.06) |  |  |  |
|  |  | AA | 23/77 | 1.39 (0.76-2.57) |  |  |  | 4/23 | 1.06 (0.34-3.28) |  |  |  |
| CYP24A1 | rs4809959 | GG | 32/133 | 1.00 | 0.99 | 1.00 |  | 14/131 | 1.00 | 0.01 | 0.45 | 0.07 |
|  |  | GA | 64/223 | 1.13 (0.7-1.83) |  |  |  | 26/172 | 1.43 (0.71-2.88) |  |  |  |
|  |  | AA | 25/98 | 0.98 (0.54-1.78) |  |  |  | 19/66 | 2.71 (1.25-5.86) |  |  |  |
| CYP24A1 | rs2245153 | AA | 45/166 | 1.00 | 0.89 | 1.00 |  | 32/194 | 1.00 | 0.17 | 1.00 | 0.37 |
|  |  | AG | 55/208 | 0.97 (0.62-1.53) |  |  |  | 20/139 | 0.85 (0.46-1.57) |  |  |  |
|  |  | GG | 14/49 | 1.1 (0.55-2.2) |  |  |  | 1/25 | 0.22 (0.03-1.72) |  |  |  |
| CYP24A1 | rs2585428 | GG | 27/109 | 1.00 | 0.65 | 1.00 |  | 26/90 | 1.00 | 0.006 | 0.17 | 0.02 |
|  |  | GA | 66/246 | 1.12 (0.67-1.87) |  |  |  | 22/175 | 0.46 (0.24-0.87) |  |  |  |
|  |  | AA | 28/100 | 1.15 (0.63-2.1) |  |  |  | 11/105 | 0.36 (0.17-0.79) |  |  |  |
| CYP24A1 | rs6022999 | GG | 15/55 | 1.00 | 0.58 | 1.00 |  | 37/200 | 1.00 | 0.33 | 1.00 | 0.87 |
|  |  | GA | 57/207 | 0.89 (0.46-1.72) |  |  |  | 17/132 | 0.73 (0.39-1.36) |  |  |  |
|  |  | AA | 49/192 | 0.83 (0.42-1.63) |  |  |  | 5/38 | 0.73 (0.26-2) |  |  |  |

*Footnote*: Odds ratio (OR) and 95% confidence interval (CI) are adjusted for covariates including age, proportion of European ancestry, body mass index, family history of breast cancer, and education. P­trend was for genetic dose-response by coding genotypes as 0, 1 and 2 based on the number of variant allele. Pinteraction was for the differences in odds ratios between pre- and postmenopausal women.

**Supplementary Table S5. SNPs in *VDR*** that show differential associations with breast cancer stratified by menopausal status in African American and European American women

| **Race** | **SNP** | **Genotype** | **Premenopausal women** | | |  | **Postmenopausal women** | | | **Pinteraction** |
| --- | --- | --- | --- | --- | --- | --- | --- | --- | --- | --- |
|  |  |  | **# case /control** | **Adjusted OR (95% CI)** | **Ptrend** |  | **# case /control** | **Adjusted OR (95% CI)** | **Ptrend** |  |
| AA | rs886441 | AA | 114/110 | 1.00 | 0.004 |  | 73/72 | 1.00 | 0.92 | 0.0005 |
|  |  | AG | 163/126 | 1.29 (0.90-1.85) |  |  | 106/96 | 1.00 (0.63-1.57) |  |  |
|  |  | GG | 59/25 | 2.27 (1.32-3.90) |  |  | 31/30 | 0.96 (0.51-1.82) |  |  |
| EA | rs7975232 (Apa1) | GG | 88/82 | 1.00 | 0.64 |  | 53/80 | 1.00 | 0.01 | 0.10 |
|  |  | GA | 103/97 | 1.01 (0.66-1.55) |  |  | 53/55 | 1.39 (0.81-2.39) |  |  |
|  |  | AA | 44/38 | 1.16 (0.67-2.03) |  |  | 40/30 | 2.24 (1.19-4.21) |  |  |

*Footnote*: Odds ratio (OR) and 95% confidence interval (CI) are adjusted for covariates including age, proportion of European ancestry, body mass index, family history of breast cancer, and education. P­trend was for genetic dose-response by coding genotypes as 0, 1 and 2 based on the number of variant allele. Pinteraction was for the differences in odds ratios between pre- and postmenopausal women, and Pinteraction < 0.10 was deemed significant.
